# NADPH oxidase in B cells and macrophages protects against murine lupus by regulation of TLR7

**DOI:** 10.1172/jci.insight.178563

**Published:** 2024-07-23

**Authors:** Rachael A. Gordon, Haylee A. Cosgrove, Anthony Marinov, Sebastien Gingras, Jeremy S. Tilstra, Allison M. Campbell, Sheldon I. Bastacky, Michael Kashgarian, Andras Perl, Kevin M. Nickerson, Mark J. Shlomchik

**Affiliations:** 1 Department of Immunology and; 2 Department of Medicine, University of Pittsburgh School of Medicine, Pittsburgh, Pennsylvania, USA.; 3 Department of Immunobiology, Yale University School of Medicine, New Haven, Connecticut, USA.; 4 Department of Pathology, University of Pittsburgh School of Medicine, Pittsburgh, Pennsylvania, USA.; 5 Department of Pathology, Yale University School of Medicine, New Haven, Connecticut, USA.; 6 Departments of Medicine, Microbiology and Immunology, Biochemistry and Molecular Biology, State University of New York, Upstate Medical University, College of Medicine, Syracuse, New York, USA.

**Keywords:** Autoimmunity, Lupus

## Abstract

Loss of NADPH oxidase (NOX2) exacerbates systemic lupus erythematosus (SLE) in mice and humans, but the mechanisms underlying this effect remain unclear. To identify the cell lineages in which NOX2 deficiency drives SLE, we employed conditional KO and chimeric approaches to delete *Cybb* in several hematopoietic cell lineages of MRL.Fas*^lpr^* SLE-prone mice. Deletion of *Cybb* in macrophages/monocytes exacerbated SLE nephritis, though not to the degree observed in the *Cybb* global KOs. Unexpectedly, the absence of *Cybb* in B cells resulted in profound glomerulonephritis and interstitial nephritis, rivaling that seen with global deletion. Furthermore, we identified that NOX2 is a key regulator of TLR7, a driver of SLE pathology, both globally and specifically in B cells. This is mediated in part through suppression of TLR7-mediated NF-κB signaling in B cells. Thus, NOX2’s immunomodulatory effect in SLE is orchestrated not only by its function in the myeloid compartment, but through a pivotal role in B cells by selectively inhibiting TLR7 signaling.

## Abstract 2

NOX2 deficiency exacerbates lupus but the cell types and mechanisms by which NOX2 exerts protective effects remain unknown. We show that NOX2 regulates lupus by its function in macrophages/monocytes and B cells, where it modulates TLR7 signaling.

## Graphical abstract

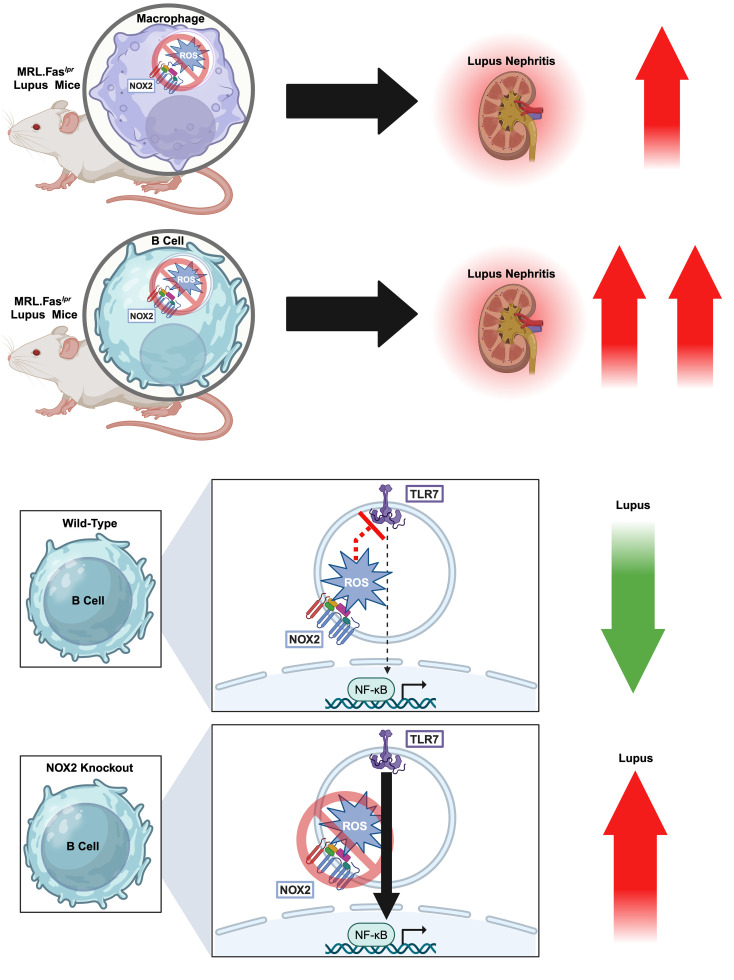

## Introduction

Systemic lupus erythematosus (SLE) is a multisystem autoimmune disease marked by loss of tolerance to self and rampant immune activation, resulting in damage to target organs (1). A hallmark feature of SLE is loss of tolerance to nuclear antigens and the formation of autoantibodies against nucleic acids and nucleoprotein complexes. The origins of autoantigens in SLE remain uncertain. Antigenic contents from dying neutrophils and/or a failure to dispose of cellular debris by macrophages are 2 possible sources of autoantigen in SLE (2). The NADPH oxidase complex, a group of transmembrane and cytosolic enzymes responsible for the respiratory burst crucial to eliminate microbes (3–5), is important for both of these processes (6–10).

Chronic granulomatous disease (CGD) results from loss-of-function mutations in key components of NADPH oxidase 2 (NOX2). Male patients with X-linked CGD have a propensity to develop a lupus-like disease (11, 12). Susceptibility to systemic autoimmunity in patients with CGD is likely to be directly mediated by lack of NAPDH oxidase activity rather than indirect effects of having CGD per se, as heterozygous female carriers of the X-linked cytochrome b-245, β polypeptide (*Cybb*) null allele also have a higher likelihood of developing SLE, despite not having CGD (13, 14). Moreover, loss-of-function polymorphisms in 2 other NOX2 components, neutrophil cytosolic factor 1 and 2 (*NCF1* and -*2*)*,* confer increased SLE susceptibility (15–18). Others and we have consistently demonstrated that multiple rodent models of CGD exhibit the same increased susceptibility to autoimmunity as observed in human patients (19–27). Thus, NOX2 is a critical immune regulator in humans and mice.

However, given its broad expression, mechanisms by which NOX2 constrains inflammation in vivo are unknown. NOX2 could inhibit inflammation by acting on neutrophils (7, 28), macrophages (7–10, 27, 29), dendritic cells (DCs) (27, 30), and T cells (31–34). NOX2 is also expressed in B cells, with proposed functions in B cell receptor (BCR) signaling, antibody production, and antigen presentation (35–40).

In addition to cell specificity, it also remains unclear how NOX2 promotes its protective effect mechanistically. Interestingly, in our aforementioned study demonstrating exacerbated SLE in *Cybb*-deficient mice, we observed an increase in anti-RNA and anti-Smith (anti-Sm) autoantibody titers (20). The formation of these autoantibodies is TLR7 dependent in a B cell–intrinsic fashion (41). Thus, it is plausible that NOX2 and TLR7 may have a regulatory interaction in SLE, and that this relationship could be B cell intrinsic.

To investigate how NOX2 deficiency promotes autoimmunity in various cell subsets, we used in vivo chimeric and conditional KO approaches to delete *Cybb*, the gene encoding a critical subunit of the NOX2 complex, in specific cell subsets of MRL.Fas*^lpr^* SLE-prone mice, a strain in which we previously demonstrated strong regulatory activity of NOX2 via global KO of *Cybb* (20). The MRL.Fas*^lpr^* mouse is a leading spontaneous model for the study of SLE, developing nearly all features of the human disease (42). Moreover, the MRL.Fas*^lpr^* strain has accurately predicted responses in human translational studies, validating its utility (42–48).

Here, we found that *Cybb* deficiency in the hematopoietic compartment decreased survival, exacerbated SLE nephritis, and altered the autoantibody response in the MRL.Fas*^lpr^* model of SLE. Furthermore, the absence of CYBB in either B cells or macrophages/monocytes drove clinical and immunologic features of SLE, with the effect in B cells being particularly strong, whereas no effects were seen upon deleting *Cybb* in neutrophils or T cells. We also demonstrate that global *Tlr7* deficiency is sufficient to suppress several hallmarks of SLE pathogenesis in *Cybb*-deficient MRL.Fas*^lpr^* mice. Finally, we show that NOX2 is a key negative regulator of TLR7 in B cells at both the genetic and signaling level, linking this mechanism to the in vivo phenotype of NOX2 deficiency. The identification of macrophages and, unexpectedly, B cells, as key sites of action for NOX2 in regulating autoimmunity represent important insights into how the NOX2 complex regulates multiple inflammatory states.

## Results

### Hematopoietic Cybb deficiency decreased survival, exacerbated kidney disease, and altered the anti-self response in SLE-prone MRL.Fas^lpr^ mice.

To determine whether NOX2 in the hematopoietic or stromal compartment is the primary driver of the exacerbated manifestations of autoimmunity that we and others observed in global NOX2-deficient mice (19–23, 26, 27), we first generated bone marrow (BM) chimeras in MRL.Fas*^lpr^* SLE-prone mice using *Cybb-*KO or control *Cybb*-sufficient donor BM. SLE pathology was assessed in chimeric mice 16–18 weeks after irradiation unless otherwise indicated.

**Figure 1:**
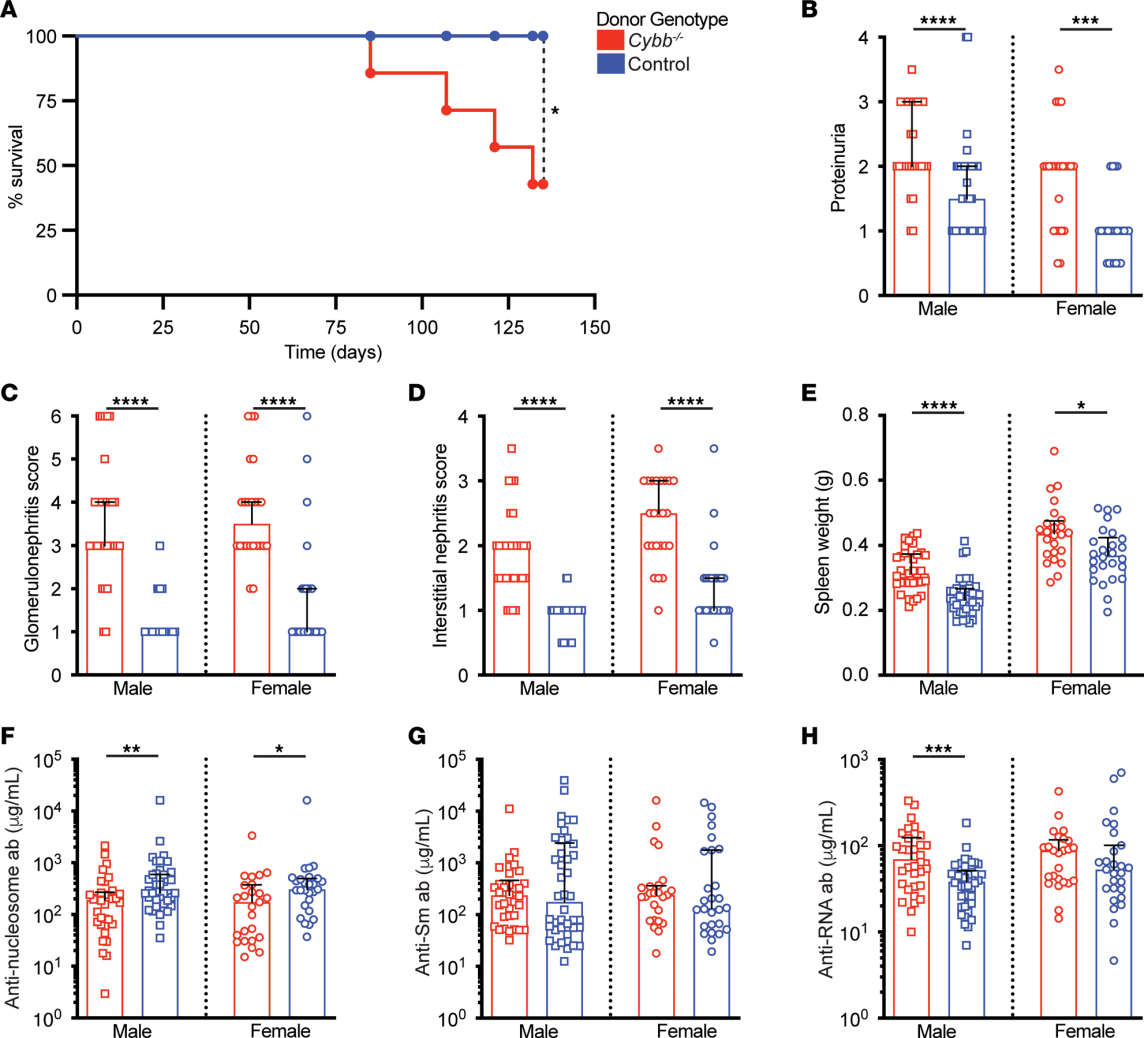
Hematopoietic *Cybb* deficiency is sufficient to decrease survival, drive nephritis, and alter the anti-self response in SLE-prone mice. Bone marrow (BM) chimeras were generated in WT MRL.Fas*^lpr^* recipients with either *Cybb*-sufficient or -deficient BM. (**A**) Kaplan-Meier survival curves for female MRL.Fas*^lpr^* BM chimeras reconstituted with BM of indicated genotypes. A log-rank test was used to determine statistical significance (donor genotype: controls *n* = 8; *Cybb^–/–^* *n* = 7 mice per group). (**B**) Proteinuria scores (donor genotype: control males *n* = 40; *Cybb^–/Y^* males *n* = 30; control females *n* = 25; *Cybb^–/–^* females *n* = 24). (**C**) Glomerulonephritis scores, (**D**) interstitial nephritis scores, and (**E**) spleen weight (donor genotype: control males *n* = 40; *Cybb^–/Y^* males *n* = 33; control females *n* = 26; *Cybb^–/–^* females *n* = 24). (**F**–**H**) Serum anti-nucleosome (**F**), anti-Sm (**G**), and anti-RNA (**H**) antibody titers (donor genotype: control males *n* = 42; *Cybb^–/Y^* males *n* = 34; control females *n* = 27; *Cybb^–/–^* females *n* = 25). Disease parameters are represented as a function of *Cybb* donor genotype. MRL.Fas*^lpr^* chimeras were evaluated 16–18 weeks after irradiation. Bars represent the median ± interquartile range (IQR). A Mann-Whitney *U* test was performed to determine statistical significance within each sex unless otherwise indicated. A Fisher’s exact test was performed to determine statistical significance for anti-Sm titers in MRL.Fas*^lpr^* mice. *P < 0.05, **P < 0.01, ***P < 0.001, ****P < 0.0001.

Strikingly, mice receiving *Cybb-*deficient BM had reduced survival and increased urine protein compared with controls (Figure 1, A and B). Mice that received *Cybb*-deficient BM also had worse glomerular and interstitial nephritis (Figure 1, C and D). Spleen weight was increased in these mice (Figure 1E). Anti-nucleosome antibody titers were reduced in SLE-prone mice with a hematopoietic *Cybb* defect (Figure 1F), consistent with altered anti-nuclear antibody patterns seen in global *Cybb-*deficient mice (20). Hematopoietic *Cybb* deficiency did not alter anti-Sm titers (Figure 1G), unlike the global *Cybb*-KO mice. Chimeras that lacked CYBB in the hematopoietic compartment had elevated anti-RNA titers in male, but not female, mice (Figure 1H).

The percentages of splenic CD11b^+^Ly6G^+^ neutrophils and CD11b^+^F4/80^+^Gr1^lo/int^ macrophages were increased in both female and male SLE-prone recipients reconstituted with *Cybb*-KO BM (Supplemental Table 1; supplemental material available online with this article; https://doi.org/10.1172/jci.insight.178563DS1), as also observed in globally *Cybb-*deficient MRL.Fas*^lpr^* mice (20).

While the aforementioned data highlight a role for hematopoietic NOX2 in regulating SLE pathogenesis, these findings do not preclude a contribution from the stromal compartment. To address this consideration, we generated reciprocal BM chimeras. Stromal *Cybb* deficiency did not drive SLE kidney disease (Supplemental Figure 1, A–C) or alter the anti-self response (Supplemental Figure 1, D–F).

### Global Tlr7 deficiency rescues exacerbated SLE in Cybb-deficient mice.

**Figure 2:**
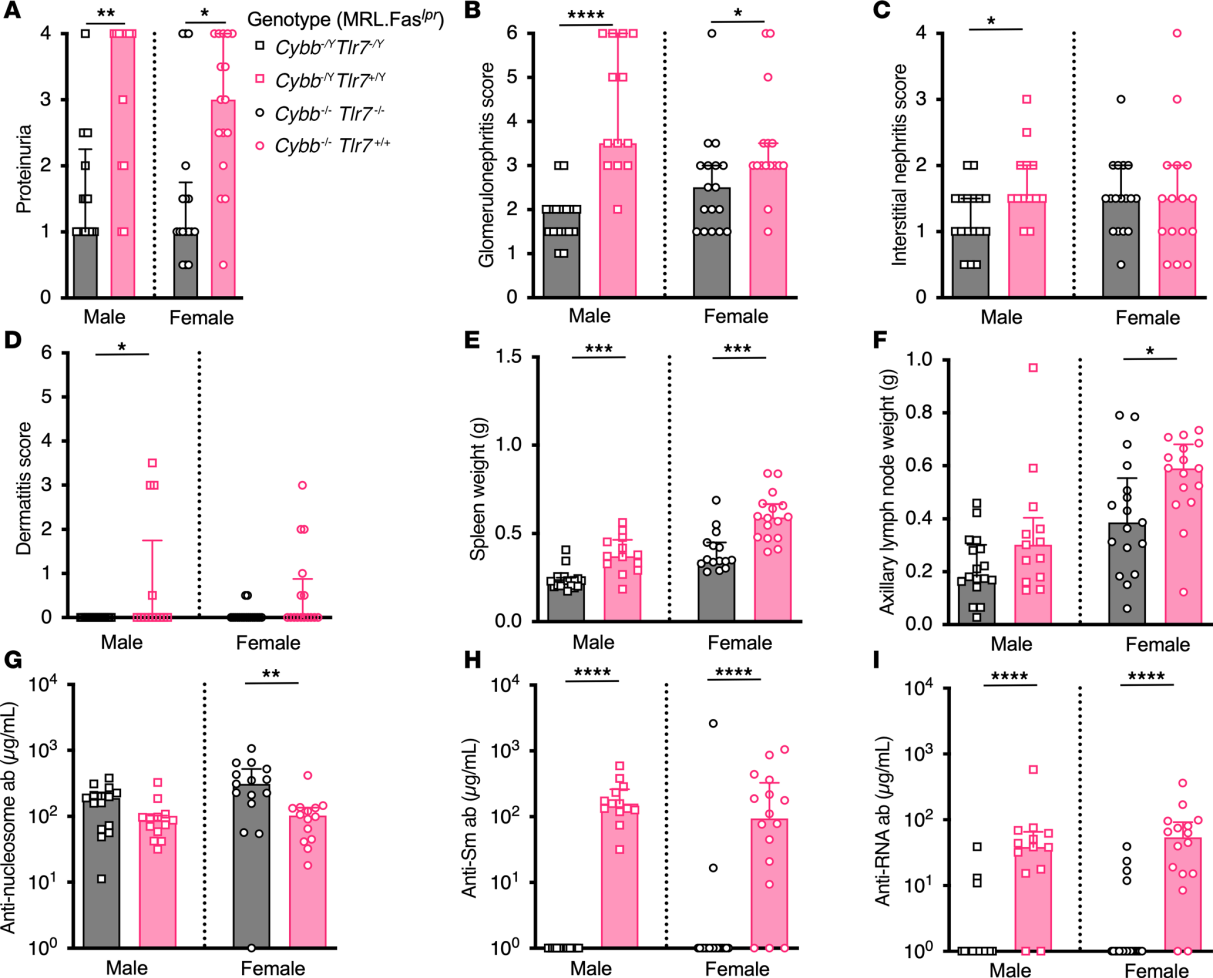
Global *Tlr7* deletion ameliorates nephritis and alters the autoantibody response in *Cybb*-deficient, SLE-prone mice. (**A**) Proteinuria scores. (**B**) Glomerulonephritis scores. (**C**) Interstitial nephritis scores. (**D**) Dermatitis scores. (**E**) Spleen and (**F**) axillary lymph node weights. (**G**–**I**) Anti-nucleosome (**G**), anti-Sm (**H**), and anti-RNA (**I**) antibody titers. Data are from mice of the indicated genotypes at 15–16 weeks of age. *Cybb^–/Y^* *Tlr7^–/Y^* males *n* = 17 (**A**–**F**), *n* = 15 (**G**–**I**); *Cybb^–/Y^* *Tlr7^+/Y^* males *n* = 13 (**A**–**I**); *Cybb^–/–^* *Tlr7^–/–^* females *n* = 17 (**A**–**F**, **H**, and **I**), *n* = 15 (**G**); *Cybb^–/–^* *Tlr7^+/+^* females *n* = 16 (**A**–**F**, **H**, and **I**), *n* = 14 (**G**). Bars represent the median ± interquartile range (IQR). A Mann-Whitney *U* test was performed to determine statistical significance within each sex unless otherwise indicated. A Fisher’s exact test was performed to determine statistical significance for anti-Sm titers in MRL.Fas*^lpr^* mice. *P < 0.05, **P < 0.01, ***P < 0.001, ****P < 0.0001.

NOX2-deficient animals have higher levels of TLR7-dependent autoantibodies. To test the hypothesis that NOX2 regulates SLE by dampening TLR7-mediated autoimmunity, we crossed globally *Tlr7-*deficient mice with global *Cybb*-KO mice on the MRL.Fas*^lpr^* SLE-prone background. At 15–16 weeks of age, compared with *Cybb*-KO mice, *Tlr7* and *Cybb* double-KO mice had significantly reduced proteinuria and glomerulonephritis (Figure 2, A and B). Interstitial nephritis was significantly decreased in male *Tlr7* and *Cybb* double-KO mice (Figure 2C). Male *Cybb^–/Y^* *Tlr7^–/Y^* mice had improved dermatitis (Figure 2D). *Tlr7* and *Cybb* double-KO mice had reduced splenomegaly, but only female mice had improved lymphadenopathy (Figure 2, E and F). As expected, *Tlr7* and *Cybb* double-KO mice had fewer TLR7-driven anti-RNA and anti-Sm autoantibodies, while female mice had increased titers of anti-nucleosome autoantibodies (Figure 2, G–I).

Compared with *Cybb-*KO controls, *Tlr7* and *Cybb* double-KO mice had reduced splenic B cell lymphopenia, a hallmark of severe SLE in both mice and humans; this was driven by an increase in the proportion of follicular B cells (Supplemental Table 1).The proportion of splenic CD11b^+^CD11c^+^ age-associated B cell–like (ABC-like) B cells was increased in females, while the proportion of plasmablasts was unchanged. The proportion of naive CD4^+^ T cells trended upwards in male mice (*P* = 0.06). The percentage of plasmacytoid DCs (pDCs) was increased in both male and female *Tlr7* and *Cybb* double-KO mice. The remainder of the myeloid compartment, including macrophages, monocytes, conventional DCs (cDCs), and neutrophils, was unchanged except for a decrease in macrophages in male mice.

### Cybb deletion in MRP8-expressing cells (neutrophils) does not impact murine SLE.

To determine in which cell types NOX2 exerts a protective effect in systemic autoimmunity, we employed a conditional KO approach to selectively delete *Cybb* in immune cell types of interest. To accomplish this, we generated a *Cybb* conditional KO allele (*Cybb^fl/fl^*), which was made directly on the MRL.Fas*^lpr^* background, using CRISPR/Cas9 technology (49). Deletion efficiency in all conditional KO strains was confirmed by qPCR of genomic DNA isolated from FACS-purified immune cell populations (Supplemental Table 2).

As *Cybb* is highly expressed in neutrophils and NOX2 has well characterized immunoregulatory functions in these cells (6–9, 29), we crossed the *Cybb^fl/fl^* allele to neutrophil-targeting *MRP8-Cre* MRL.Fas*^lpr^* mice (50, 51), generating a cohort of experimental *Cybb^fl/fl^ MRP8-Cre^+/–^* and Cre-negative *Cybb^fl/fl^* littermate controls. SLE phenotypes were assessed at 18–20 weeks of age. *Cybb* was efficiently deleted in splenic CD11b^+^Gr1^+^ neutrophils isolated from *Cybb^fl/Y^* *MRP8-Cre^+/–^* males (89.4% ± 0.8% of alleles deleted) and *Cybb^fl/fl^ MRP8-Cre^+/–^* females (88.3% ± 0.8% of alleles deleted) (Supplemental Table 2).

**Figure 3:**
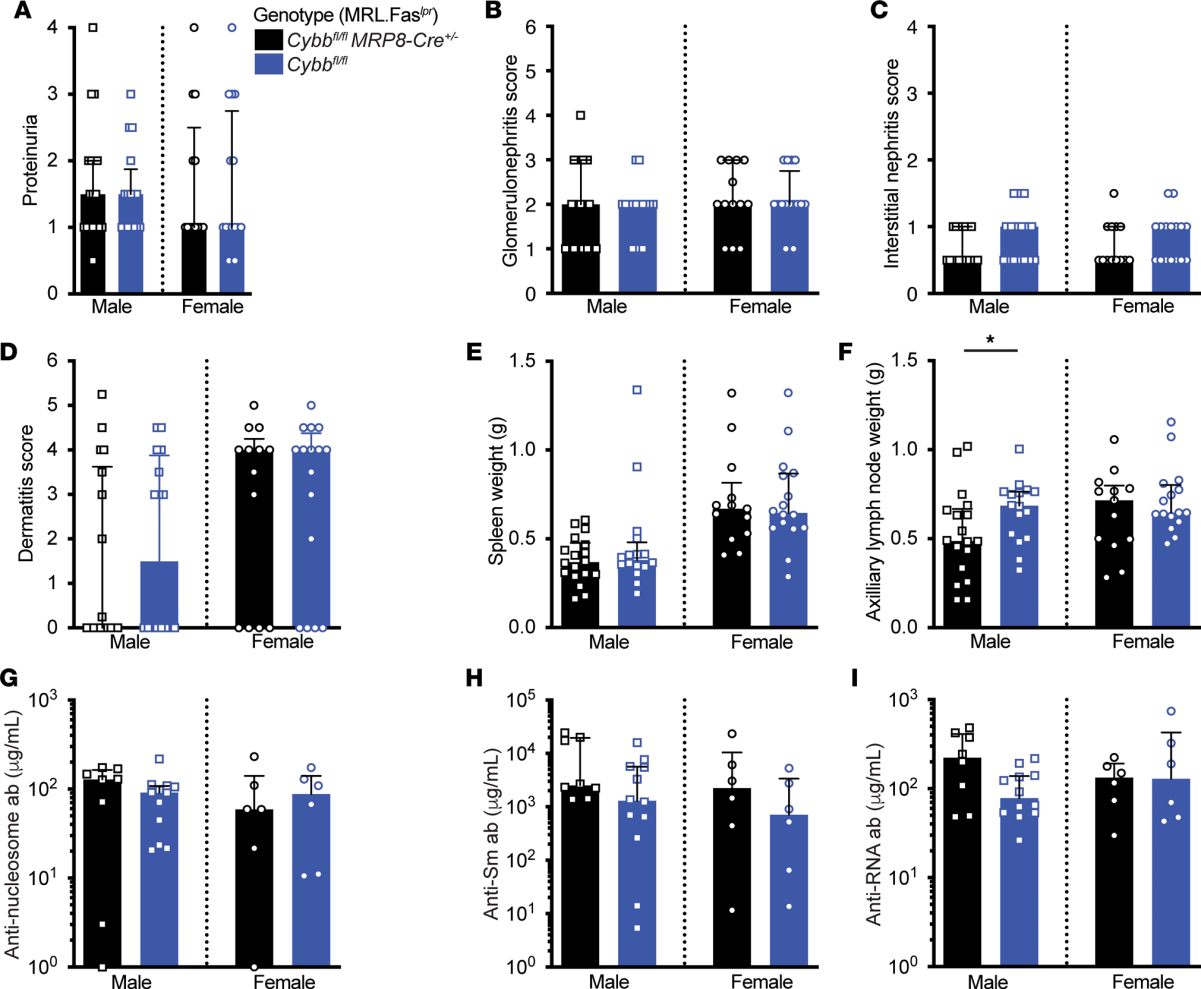
Conditional deletion of *Cybb* in neutrophils does not impact murine SLE. (**A**) Proteinuria scores. (**B**) Glomerulonephritis scores. (**C**) Interstitial nephritis scores. (**D**) Dermatitis scores. (**E**) Spleen and (**F**) axillary lymph node weights. (**G**–**I**) Anti-nucleosome (**G**), anti-Sm (**H**), and anti-RNA (**I**) antibody titers. Cohorts were assessed at 18–20 weeks of age. For the *Cybb^fl/fl^* *MRP8-Cre* cohort, the following sample sizes were used: *Cybb^fl/Y^* males *n* = 16 (**A**–**F**), *n* = 12 (**G**–**I**); *Cybb^fl/Y^ MRP8-Cre^+/–^* males *n* = 18 (**A**–**F**), *n* = 8 (**G**–**I**); *Cybb^fl/fl^* females *n* = 16 (**A**–**F**), *n* = 6 (**G**–**I**); *Cybb^fl/fl^ MRP8-Cre^+/–^* females *n* = 13 (**A**–**F**), *n* = 6 (**G**–**I**). Bars represent the median ± interquartile range (IQR). A Mann-Whitney *U* test was performed to determine statistical significance within each sex unless otherwise indicated. A Fisher’s exact test was performed to determine statistical significance for anti-Sm titers in MRL.Fas*^lpr^* mice. *P < 0.05.

We did not observe any differences in proteinuria, nephritis, dermatitis, or splenomegaly in the *Cybb^fl/fl^ MRP8-Cre* cohort (Figure 3, A–E). Axillary lymph node weights were increased in male controls, but this finding was not seen in any of the other control cohorts (Figure 3F). *Cybb* deficiency in neutrophils did not impact titers of anti-nucleosome, -Sm, or -RNA autoantibodies (Figure 3, G–I). The percentages of splenic T cells, B cells, macrophages, neutrophils, cDCs, and pDCs were similar across genotypes (Supplemental Table 1).

### Cybb deletion in LysM-expressing cells (macrophages/monocytes and neutrophils) exacerbates nephritis.

To test the roles of NOX2 in macrophages/monocytes, we generated homozygous *Cybb^fl/fl^* mice that were also heterozygous for *LysM-Cre* (51, 52). SLE phenotypes were assessed at 18–20 weeks of age. *LysM-Cre* is expressed in both neutrophils and macrophages/monocytes (53). As *Cybb* deletion in neutrophils did not alter clinical or immunologic parameters of SLE, we can deduce that any phenotype observed in the *Cybb^fl/fl^ LysM-Cre* cohort is driven by the function of NOX2 in macrophages/monocytes. While *Cybb* was efficiently deleted in CD11b^+^Gr1^+^ splenic neutrophils from *Cybb^fl/fl^ LysM-Cre* mice, *Cybb* deletion in splenic CD11b^+^F4/80^+^ macrophages was 27% ± 6% of alleles in *Cybb^fl/Y^ LysM-Cre^+/–^* males and 43% ± 6% of alleles in *Cybb^fl/fl^ LysM-Cre^+/–^* females (Supplemental Table 2).

**Figure 4:**
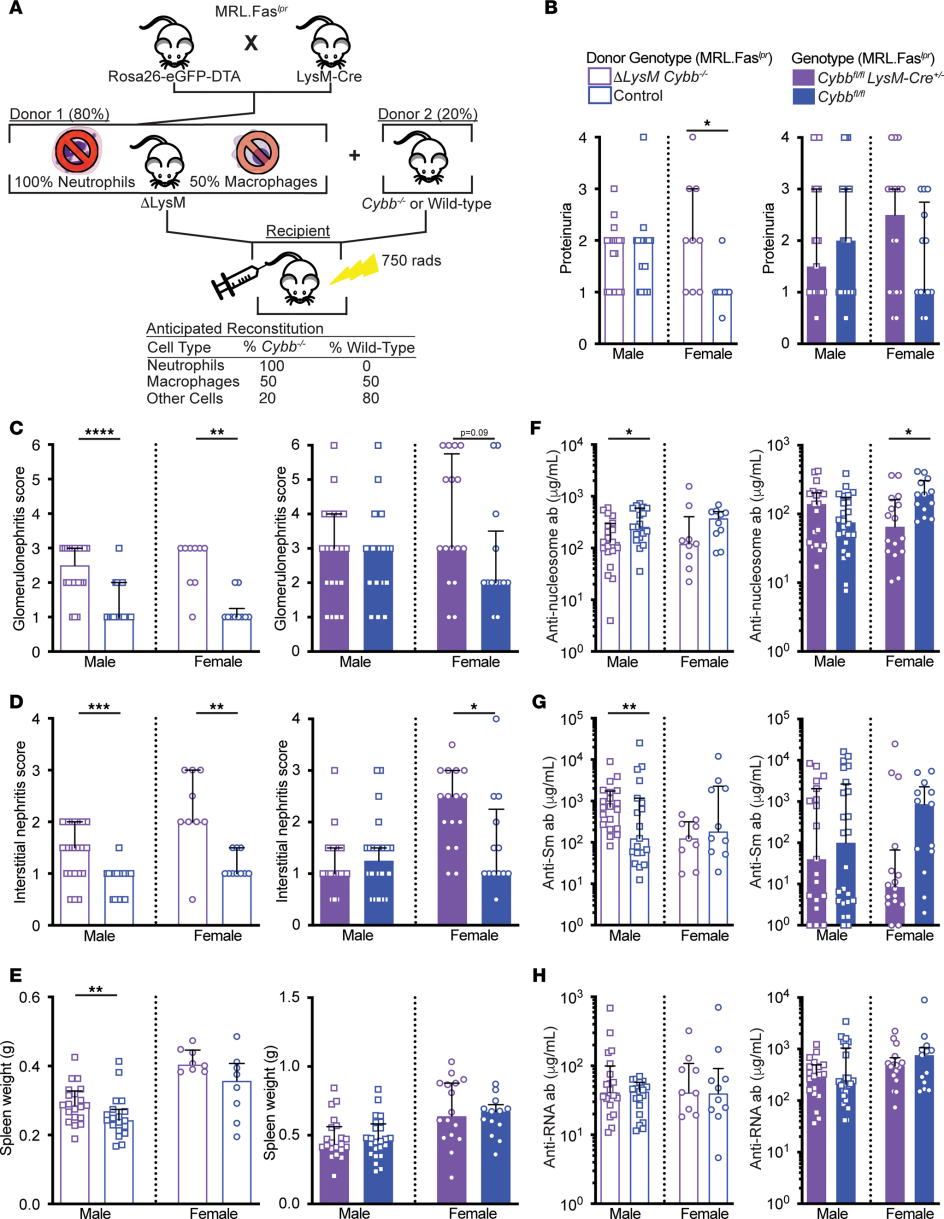
The absence of *Cybb* in monocytes/macrophages partially drives exacerbated kidney disease in SLE-prone mice. (**A**) Experimental design. Mixed BM chimeras were generated by reconstituting irradiated MRL.Fas*^lpr^* recipients with *Rosa26-eGFP-DTA*^–/+^ *LysM Cre^+/–^* (ΔLysM) and *Cybb*-sufficient or -deficient BM at an 80:20 ratio. Chimeric mice were aged for 16 weeks after irradiation. Conditional KO cohorts were aged 18–20 weeks. (**B**) Proteinuria scores. (**C**) Glomerulonephritis scores. (**D**) Interstitial nephritis scores. (**E**) Spleen weights. (**F**–**H**) Anti-nucleosome (**F**), anti-Sm (**G**), and anti-RNA (**H**) antibody titers. ΔLysM *Cybb^–/Y^* males *n* = 21 (**B**), *n* = 22 (**C** and **D**), *n* = 20 (**E**–**H**); control males *n* = 21 (**B**–**D**) and *n* = 19 (**E**–**H**); ΔLysM *Cybb^–/–^* females *n* = 9 (**B**–**D**, and **F**–**H**), *n* = 8 (**E**); control females *n* = 9 (**B**), *n* = 10 (**C** and **D**, and **F**–**H**), *n* = 8 (**E**). The sample sizes for each group within the *Cybb^fl/fl^* *LysM-Cre* cohort were the following: *Cybb^fl/Y^* males *n* = 21 (**B**–**E**), *n* = 19 (**F**–**H**); *Cybb^fl/Y^ LysM-Cre^+/–^* males *n* = 22 (**B**), *n* = 24 (**C**–**H**); *Cybb^fl/fl^* females *n* = 13; *Cybb^fl/fl^ LysM-Cre^+/–^* females *n* = 16. Bars represent the median ± interquartile range (IQR). A Mann-Whitney *U* test was performed to determine statistical significance within each sex unless otherwise indicated. A Fisher’s exact test was performed to determine statistical significance for anti-Sm titers in MRL.Fas*^lpr^* mice. *P < 0.05, **P < 0.01, ***P < 0.001, ****P < 0.0001.

Although we did not observe any significant differences in proteinuria among the groups, interstitial nephritis was exacerbated in *Cybb^fl/fl^ LysM-Cre^+/–^* female mice and there was a trend toward worse glomerulonephritis (Figure 4, B–D). No differences in splenomegaly were observed (Figure 4E). While female *Cybb^fl/fl^* *LysM-Cre^+/–^* mice had reduced titers of anti-nucleosome antibodies (Figure 4F), no differences in anti-Sm or anti-RNA titers were observed (Figure 4, G and H). *Cybb^fl/fl^* *LysM-Cre^+/–^* females had elevated frequencies of CD11b^+^Gr1^+^ splenic neutrophils. The male *Cybb^fl/Y^* *LysM-Cre^+/–^* cohort had increased percentages of total B cells and antibody-forming cells (AFCs). No other differences in the myeloid, B cell, or T cell compartments were observed between the groups (Supplemental Table 1).

The mild exacerbation of disease seen in *Cybb^fl/fl^ LysM-Cre* mice did not fully reproduce the exacerbated disease that we observed in the *Cybb* global KO (20), global heterozygote, or BM chimera cohorts. One explanation for this finding is that *Cybb* deletion efficiency was only 27% and 43% among splenic macrophages in male and female conditional KO cohorts, respectively (Supplemental Table 2), thus muting any potential phenotype. Therefore, to more thoroughly explore the role of *Cybb* in the myeloid compartment, we generated mixed BM chimeras with 80% of BM from the *Rosa26-EGFP-DTA^+/–^ LysM Cre^+/–^* MRL.Fas*^lpr^* strain — which cannot form cells expressing LysM (i.e., almost all neutrophils and ~50% of splenic macrophages; Figure 4A and Supplemental Methods) — and 20% of BM from *Cybb*-KO MRL.Fas*^lpr^* mice. This strategy resulted in experimental mice in which *Cybb* was deleted in approximately 88% of splenic CD11b^+^Gr1^+^ neutrophils and absent in approximately 46% of CD11b^+^F4/80^+^ splenic macrophages (Supplemental Table 2). The experimental chimeric mice are referred to as Δ*LysM Cybb^–/–^*. SLE phenotypes were assessed in chimeric mice 16 weeks after irradiation. Proteinuria was increased in Δ*LysM* *Cybb^–/–^* MRL.Fa*s^lpr^* female mice (Figure 4B). Glomerular and interstitial nephritis was exacerbated in both male and female Δ*LysM* *Cybb^–/–^* MRL.Fas*^lpr^* mice compared with controls (Figure 4, C and D). Spleen weights were increased in male, but not female, Δ*LysM* *Cybb^–/–^* MRL.Fas*^lpr^* mice (Figure 4E).

Male Δ*LysM* *Cybb^–/Y^* MRL.Fas*^lpr^* mice had reduced serum anti-nucleosome titers (Figure 4F) compared with their respective controls, but this trend did not reach significance among females, which comprised a smaller cohort. Anti-Sm titers were elevated in male Δ*LysM* *Cybb^–/Y^* mice (Figure 4G), but anti-RNA titers were similar between groups (Figure 4H). Total κ Ig AFC ELISpots were elevated in male Δ*LysM* *Cybb^–/Y^*, but not female, MRL.Fas*^lpr^* mice compared with controls (Supplemental Table 1). There were no statistically significant differences in AFC ELISpots for the IgG1, IgG2a, and IgM isotypes (Supplemental Table 1). By flow cytometry, CD19^lo/int^CD44^+^CD138^+^intracellular κ^hi^ AFCs were elevated in female Δ*LysM* *Cybb^–/–^*, but not male Δ*LysM* *Cybb^–/Y^*, mice (Supplemental Table 1).

A potential limitation of the mixed BM chimera experiment is that 20% of BM-derived cells that are not *LysM* positive also lack *Cybb*. This could be relevant since *Cybb* heterozygous (*Cybb*^+/–^) female MRL.Fas*^lpr^* mice, in which 50% of cells lacked CYBB protein due to mosaic X inactivation, developed exacerbated SLE-like disease (20), indicating that the presence of a subset of cells that lacks CYBB can have a dominant effect in promoting the disease. To rule out that the absence of CYBB in 20% of cells could mediate the exacerbated disease phenotype seen in the Δ*LysM* *Cybb^–/–^* cohort, we generated an additional control of mixed complete 80:20 wild-type (WT)/*Cybb*-KO BM chimeras. Reconstitution of WT MRL.Fas*^lpr^* recipients with 20% of *Cybb*-KO BM did not worsen renal disease (Supplemental Figure 2, A–C) or alter the autoantibody response (Supplemental Figure 2, D–F). Thus, we conclude that in the Δ*LysM* *Cybb^–/–^* cohort, the 20% of non*–LysM*-expressing cells that are *Cybb* deficient are not responsible for the observed phenotypes.

### T cell Cybb deficiency does not affect murine SLE.

To test the hypothesis that NOX2 deficiency in T cells contributed to autoimmunity in SLE, we crossed the *Cybb^fl/fl^* with *CD4-Cre* (54) MRL.Fas*^lpr^* strains to generate a cohort of experimental *Cybb^fl/fl^ CD4-Cre*–positive mice and Cre-negative *Cybb^fl/fl^* controls. Male cohorts were analyzed at 22 weeks of age and female cohorts were analyzed at 19 weeks of age. *Cybb* was efficiently deleted in both the CD4^+^ and CD8^+^ T cell compartments (Supplemental Table 2). We did not observe any significant differences in kidney disease (Supplemental Figure 3, A–C), dermatitis (Supplemental Figure 3D), or lymphoproliferation (Supplemental Figure 3, E and F). Autoantibody titers were similar between the groups, with the exception of anti-RNA levels which were decreased in female *Cybb^fl/fl^ CD4-Cre^+/–^* mice (Supplemental Figure 3, G–I).

### B cell–specific Cybb deletion results in increased nephritis and altered autoantibody responses.

To test the function of B cell–expressed NOX2 in SLE pathogenesis, we generated *Cybb^fl/fl^* *CD19-Cre^+/–^* MRL.Fas*^lpr^* mice (55, 56). *CD19-Cre*–negative *Cybb^fl/fl^* littermates served as a negative control. SLE pathology was assessed at 16–17 weeks of age. *Cybb* was efficiently deleted in CD19^+^ B cells isolated from spleens of *Cybb^fl/fl^* *CD19-Cre^+/–^* mice (85.6% ± 2.5% of alleles) (Supplemental Table 2).

**Figure 5:**
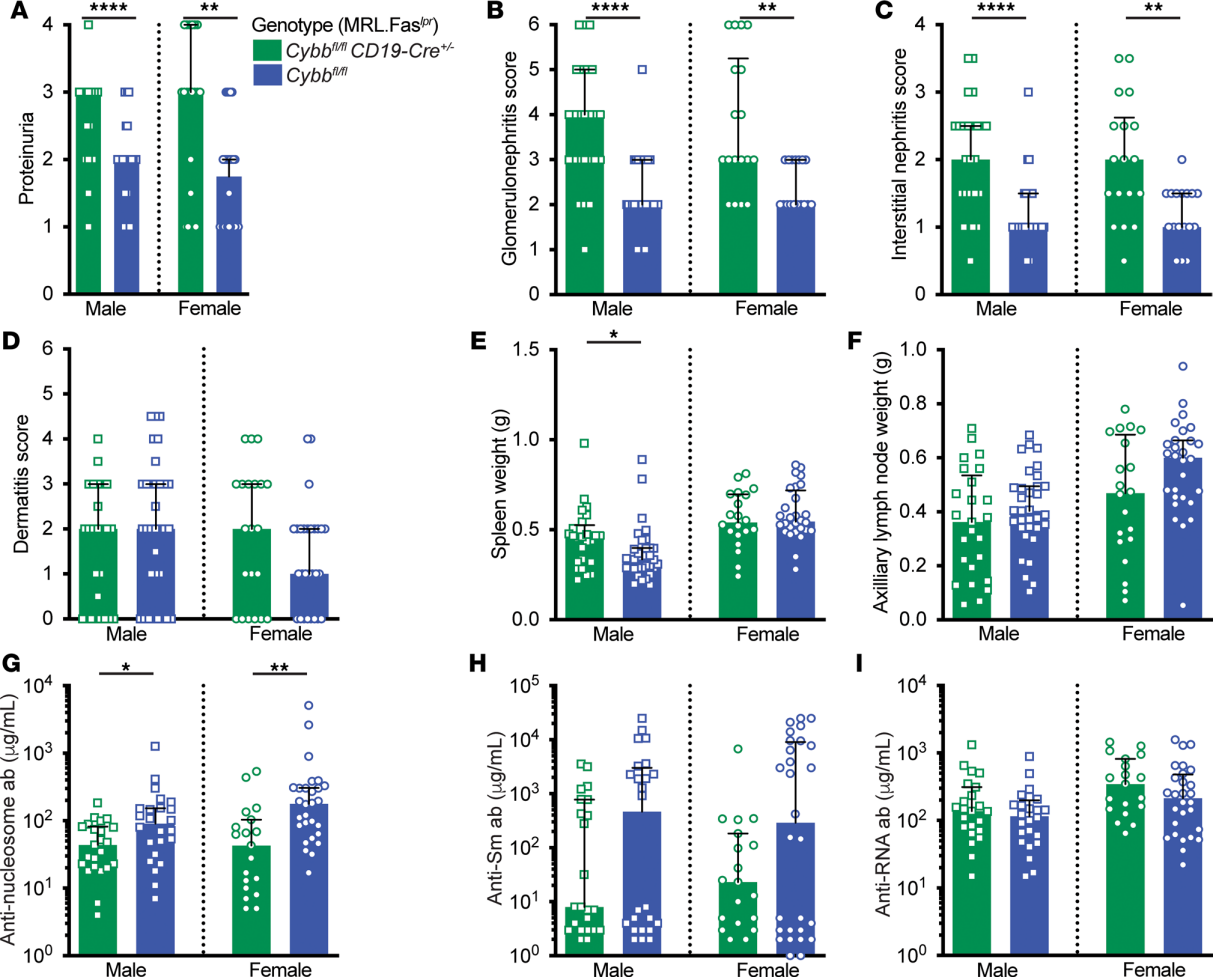
*Cybb* deletion in B cells exacerbates nephritis and alters the autoantibody response in SLE-prone mice. (**A**) Proteinuria scores. (**B**) Glomerulonephritis scores. (**C**) Interstitial nephritis scores. (**D**) Dermatitis scores. (**E**) Spleen and (**F**) axillary lymph node weights. (**G**–**I**) Anti-nucleosome (**G**), anti-Sm (**H**), and anti-RNA (**I**) antibody titers. Data are from mice of the indicated genotypes at 16–17 weeks of age. *Cybb^fl/Y^* males *n* = 33 (**A**–**F**), *n* = 24 (**G**–**I**); *Cybb^fl/Y^ CD19-Cre^+/–^* males *n* = 26 (**A** and **D**), *n* = 25 (**B**, **C**, **E**, and **F**), *n* = 23 (**G**–**I**); *Cybb^fl/fl^* females *n* = 28 (**A** and **D**–**I**), 17 (**B** and **C**); *Cybb^fl/fl^ CD19-Cre^+/–^* females *n* = 21 (**A** and **D**), *n* = 18 (**B** and **C**), *n* = 20 (**E** and **F**), *n* = 19 (**G**–**I**). Bars represent the median ± interquartile range (IQR). A Mann-Whitney *U* test was performed to determine statistical significance within each sex unless otherwise indicated. A Fisher’s exact test was performed to determine statistical significance for anti-Sm titers in MRL.Fas*^lpr^* mice. *P < 0.05, **P < 0.01, ****P < 0.0001.

Male and female *Cybb^fl/fl^* *CD19-Cre^+/–^* MRL.Fas*^lpr^* mice developed markedly increased proteinuria (Figure 5A), exacerbated glomerulonephritis, and more interstitial nephritis (Figure 5, B and C). Dermatitis severity and incidence was not different among the groups (Figure 5D). *Cybb^fl/Y^ CD19-Cre^+/–^* male, but not *Cybb^fl/fl^ CD19-Cre^+/–^* female, mice had increased spleen weights (Figure 5E). There were no differences in lymph node weights among the groups (Figure 5F).

Anti-nucleosome titers were lower in both male and female *Cybb^fl/fl^ CD19-Cre^+/–^* MRL.Fas*^lpr^* mice (Figure 5G). There were no differences in anti-RNA or anti-Sm titers among the groups (Figure 5, H and I). No differences were observed in the percentages of total CD19^+^ B cells or CD19^lo/int^CD44^+^CD138^+^intracellular κ^hi^ AFCs in *Cybb^fl/Y^ CD19-Cre^+/–^* versus control animals (Supplemental Table 1). Similarly, there were no differences in follicular, marginal zone, CD11b^+^CD11c^+^ ABCs, or germinal center B cells among the groups (Supplemental Table 1).

### B cell–intrinsic Tlr7 deficiency is sufficient to ameliorate severe nephritis in Cybb-deficient mice.

**Figure 6:**
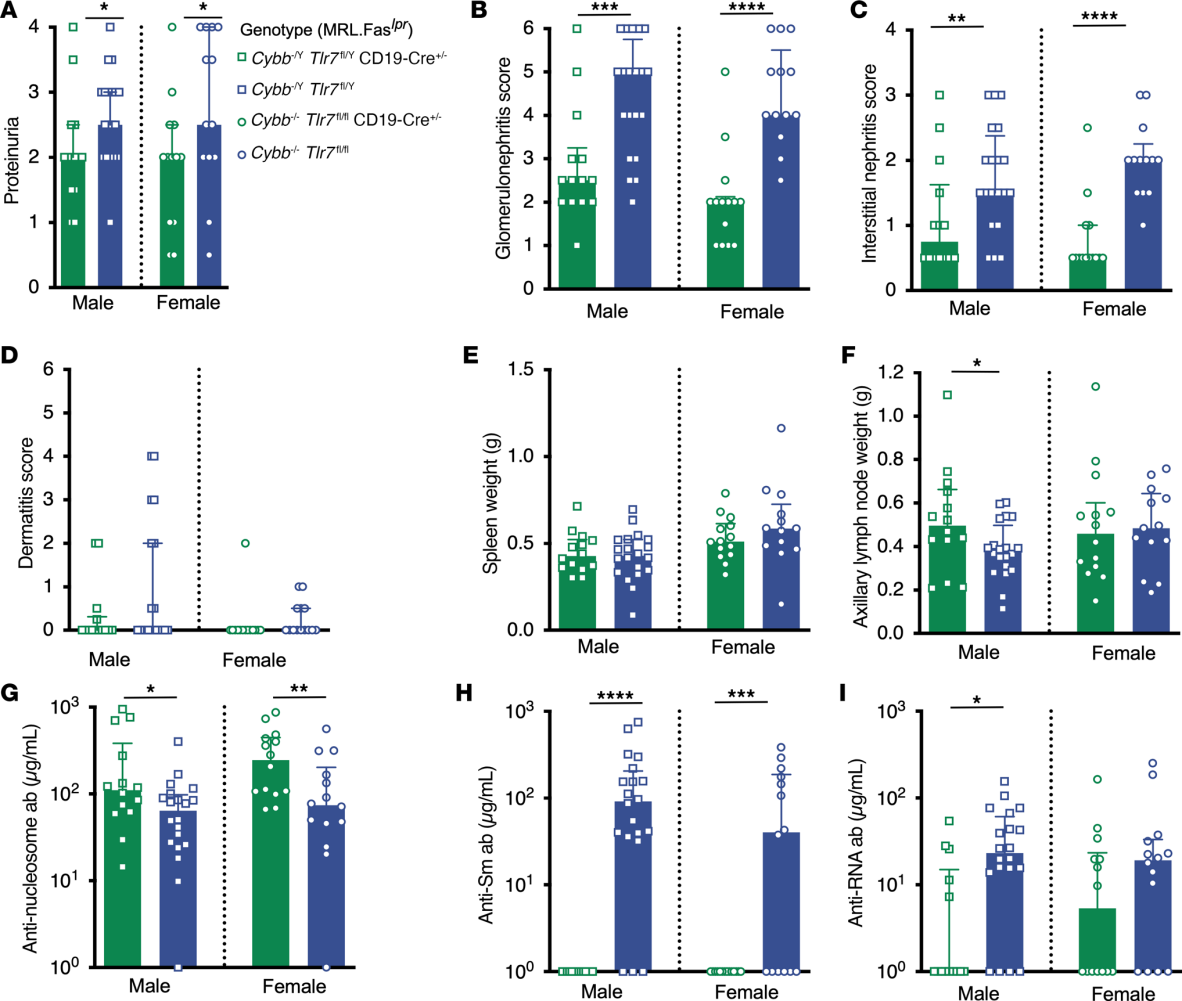
*Tlr7* deletion in B cells ameliorates nephritis and alters the autoantibody response in *Cybb*-deficient, SLE-prone mice. (**A**) Proteinuria scores. (**B**) Glomerulonephritis scores. (**C**) Interstitial nephritis scores. (**D**) Dermatitis scores. (**E**) Spleen and (**F**) axillary lymph node weights. (**G**–**I**) Anti-nucleosome (**G**), anti-Sm (**H**), and anti-RNA (**I**) antibody titers. Data are from mice of the indicated genotypes at 15–16 weeks of age. *Cybb^–/Y^* *Tlr7^fl/Y^ CD19-Cre^+/–^* males *n* = 14 (**A**–**I**); *Cybb^–/Y^* *Tlr7^fl/Y^* males *n* = 19 (**A**), *n* = 20 (**B**–**I**), *Cybb^–/–^* *Tlr7^fl/fl^ CD19-Cre^+/–^* females *n* = 13 (**A**), *n* = 14 (**B**–**I**); *Cybb^–/–^* *Tlr7^fl/fl^* females *n* = 13 (**A**), *n* = 14 (**B**–**I**). Bars represent the median ± interquartile range (IQR). A Mann-Whitney *U* test was performed to determine statistical significance within each sex unless otherwise indicated. A Fisher’s exact test was performed to determine statistical significance for anti-Sm titers in MRL.Fas*^lpr^* mice. *P < 0.05, **P < 0.01, ***P < 0.001, ****P < 0.0001.

Since global disease exacerbation mediated by the loss of NOX2 was ameliorated by global absence of TLR7, we wanted to determine in which cell types this effect was being mediated. TLR7 was previously shown to have a role in B cells in promoting disease, and here we showed (Figure 5) that NOX2 regulates disease in B cells. Hence, we hypothesized that B cell–intrinsic TLR7 would be important in driving NOX2-deregulated SLE. To test this, we deleted *Tlr7* in B cells of globally *Cybb*-deficient mice MRL.Fas*^lpr^* mice by generating *Cybb^–/Y^ Tlr7^fl/Y^ CD19-Cre^+/–^* (male) and *Cybb^–/–^ Tlr7^fl/fl^* *CD19-Cre^+/–^* (female) cohorts. SLE phenotypes were assessed at 15 weeks, comparing Cre-positive to Cre-negative littermate controls. Strikingly, all parameters of SLE, including proteinuria, glomerulonephritis, and interstitial nephritis were significantly improved in both males and females (Figure 6, A–C) when TLR7 was specifically and only deleted in B cells on a global *Cybb-*deficient background. Unlike in the cohort in which both genes were deleted globally, spleen weight and skin disease were not different from controls, while *Cybb^–/Y^ Tlr7^fl/Y^* *CD19-Cre^+/–^* males demonstrated an increase in lymphadenopathy (Figure 6, D–F). Similar to the global KO cohort, experimental mice had reduced anti-RNA and anti-Sm autoantibodies, with an increase in anti-nucleosome autoantibodies (Figure 6, G–I).

There were no differences in splenic frequencies of various immune cell types (Supplemental Table 1), except for a small increase in DC proportions and, notably, an increase in the percentage of naive CD4^+^ T cells in female mice.

### Cybb-KO B cells have increased NF-κB pathway activation after TLR7 stimulation.

**Figure 7:**
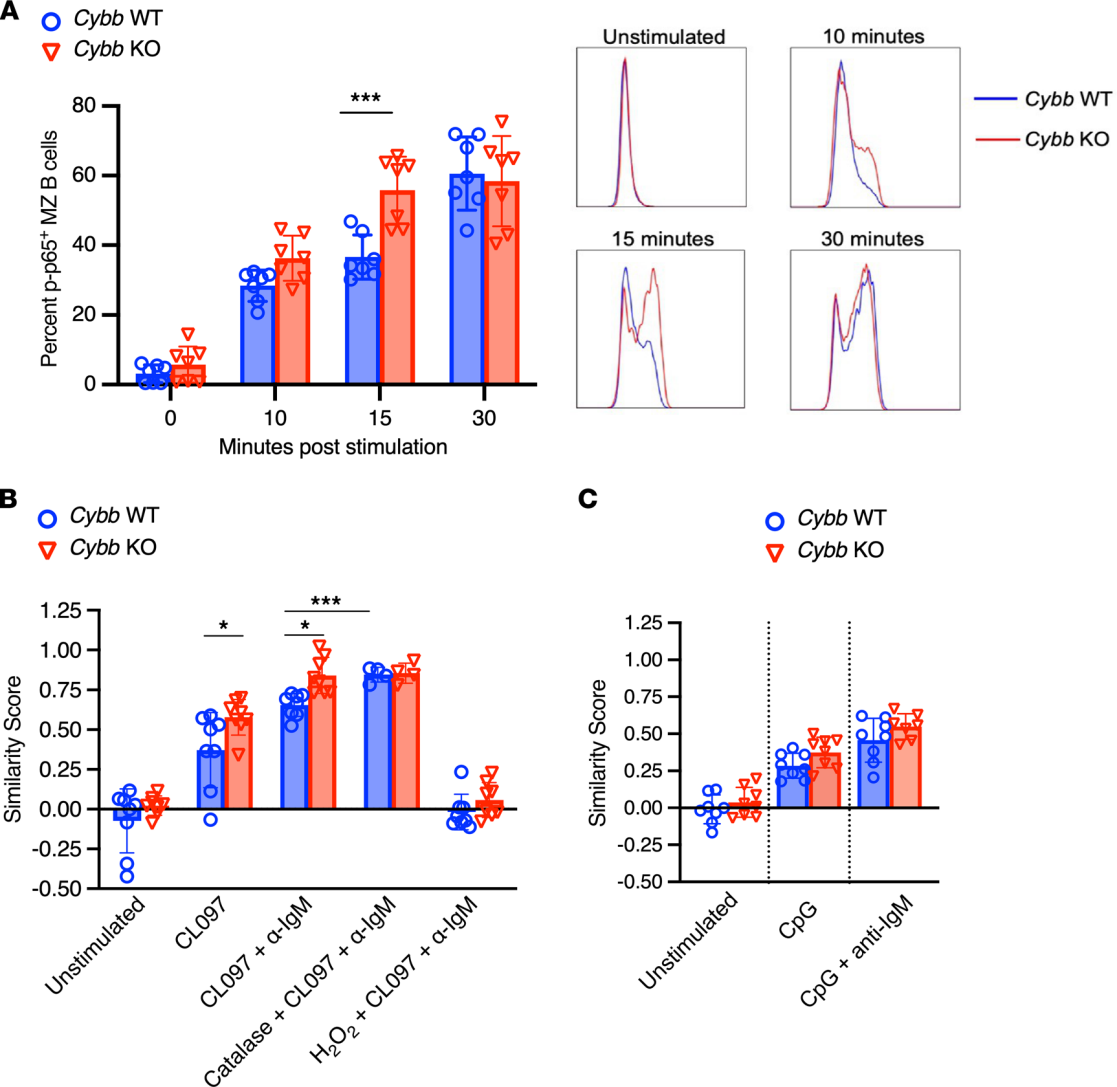
*Cybb*-knockout B cells have increased NF-κB pathway activation after TLR7 stimulation. (**A**) FACS analysis of phosphorylated p65 in C57BL/6 WT (*n* = 8, circles) and C57BL/6 *Cybb*-KO (*n* = 7, triangles) marginal zone B cells stimulated with 2.5 μg/mL TLR7 agonist CL097 plus 20 μg/mL anti-IgM (left panel) and representative flow plots showing p-p65 upregulation after CL097 plus anti-IgM stimulation (right panel). (**B**) Similarity score quantifying p65 nuclear translocation in C57BL/6 WT (circles) and C57BL/6 *Cybb*-KO (triangles) marginal zone B cells after no stimulation (*n* = 8), 2.5 μg/mL TLR7 agonist CL097 (*n* = 8), 2.5 μg/mL TLR7 agonist CL097 plus 20 μg/mL anti-IgM (*n* = 8), 1000 U/mL pretreatment with catalase followed by CL097 plus anti-IgM (*n* = 4), or 100 μM pretreatment with hydrogen peroxide followed by CL097 plus anti-IgM (*n* = 8). (**C**) Similarity score quantifying p65 nuclear translocation in C57BL/6 *Cybb* WT (circles) and C57BL/6 *Cybb*-KO (triangles) marginal zone B cells after no stimulation (*n* = 8), 10 μg/mL TLR9 agonist CpG ODN 1826 (*n* = 8), and 10 μg/mL TLR9 agonist CpG ODN 1826 plus 20 μg/mL anti-IgM (WT *n* = 8 and *Cybb*-KO *n* = 7). Scatter plots display data from individual samples from individual mice, with black lines showing median values. Two-way ANOVA with Holm-Šidák correction was used to determine statistical significance between WT and *Cybb*-KO samples; 2-tailed Student’s *t* tests were used to determine statistical significance of catalase treatment within each genotype. *P < 0.05, ***P < 0.001.

While the results presented thus far indicate that the absence of NOX2 promotes B cell–specific, TLR7-dependent autoimmunity, whether this is a direct or indirect effect in the B cell could not be distinguished. To test for a direct effect of the absence of NOX2 in promoting TLR7 signaling, we measured NF-κB signaling downstream of TLR7 activation. We analyzed both upregulation of the phosphorylation of the NF-κB subunit p65 (p-p65) as well as p65 nuclear translocation in *Cybb*-sufficient and *Cybb-*deficient marginal zone B cells; marginal zone B cells have higher levels of TLR7 (41), making them more sensitive to TLR7 stimulation and thus enhancing the sensitivity of the approach. To demonstrate the fundamental biology of this relationship, we utilized ex vivo marginal zone B cells from WT and *Cybb*-KO B6 mice, as the excessive background inflammation in MRL.Fas*^lpr^* mice, which in turn can modulate TLR7 expression levels, could have confounded the conclusions. As BCR and TLR signals synergize to activate autoreactive B cells in SLE, we stimulated marginal zone B cells with a combination of anti-IgM and CL097, a TLR7 agonist. Fifteen minutes after stimulation, we observed an increase the percentage of p-p65^+^ B cells in *Cybb*-KO cells versus WT cells (Figure 7A).

As an additional and more definitive measure of TLR7-mediated activation of the NF-κB family, we assessed p65 nuclear translocation after stimulation with anti-IgM and CL097 or with CL097 alone. Consistent with a direct negative regulatory role of NOX2 on TLR7 signaling in B cells, we observed an increase in nuclear translocation in *Cybb*-KO B cells compared with *Cybb*-sufficient controls (Figure 7B) in cells treated with BCR plus TLR7 stimulation or with TLR7 stimulation alone. Based on the established importance of cysteine residues in TLR7’s ectodomain (57, 58), which theoretically could be modulated by NOX2-derived reactive oxygen species (ROS), we also pretreated selected samples with either catalase (to breakdown ROS) or hydrogen peroxide (as a source of ROS). Interestingly, hydrogen peroxide pretreatment quenched TLR7-initiated NF-κB signaling via CL097 and anti-IgM, while catalase pretreatment did not affect signaling in *Cybb*-KO cells but caused signaling in WT cells to significantly increase (Figure 7B). Importantly, *Cybb* genotype did not affect the response to stimulation of TLR9 with CpG alone or CpG and anti-IgM stimulation, indicating that this regulatory mechanism via NOX2 appears specific to activation through TLR7 (Figure 7C).

## Discussion

The significance of NOX2 as a negative regulator of autoimmunity in multiple animal models, as well as human autoimmune syndromes, is becoming increasingly recognized (11–27). However, the mechanisms by which NOX2 mediates these effects have been largely unknown. Based on the totality of the cell-specific NOX2 deletion systems, encompassing 4 different Cre strains along with the mixed BM chimera approaches, here we have identified both B cell– and macrophage/monocyte-expressed NOX2 as fundamental negative regulators of SLE pathogenesis. In addition to identifying the cell-type-specific roles of NOX2 in our models — which by itself is important information — we have identified a B cell–intrinsic mechanism by which NOX2 operates. In B cells, NOX2 selectively regulates TLR7, but not TLR9, signaling. Thus, we have provided what we believe are new insights into major unaddressed questions regarding the emerging role of NOX2 in autoimmunity: in which cell types and by what mechanism does NOX2 mediate its regulatory functions?

A dominant regulatory role for NOX2 in the B cell was unexpected. Notably, the disease phenotype of B cell–specific *Cybb* deletion is more severe than that seen in any of the *LysM-Cre*–dependent systems, underscoring the relative importance of B cell–expressed NOX2 in the MRL.Fas*^lpr^* lupus model. In fact, B cell–specific NOX2 deletion nearly recapitulates the effects of global deletion. In line with our findings, contemporaneous work by Liu et al. showed that deletion of B cell–intrinsic *Ncf1* (which encodes a cytosolic subunit of NADPH oxidase) results in spontaneous B cell activation and increased autoantibodies in aged WT mice (59). These phenotypes were partially reproduced in low-penetrant mouse models of systemic autoimmunity (59). However, disease endpoints were not assessed in these model systems and it is well known that autoantibodies and disease do not necessarily follow, including in the case of TLR9 deletion where anti-DNA antibodies are abrogated, while disease is actually exacerbated (60, 61). Thus, our finding that NOX2 deficiency in the B cell compartment exacerbates both clinical disease (nephritis) and immunologic endpoints in a relevant spontaneous polygenic model of SLE is a distinctive and significant milestone in the understanding of NOX2 in the context of autoimmunity.

It is conceivable that NOX2 plays a crucial role in dampening the activity of a newly recognized subset of inflammatory B cells known as “atypical” or double-negative 2 memory B cells in humans, or ABCs in mice (62, 63). ABCs are found at higher frequency in patients with SLE and in mouse models of lupus (62, 63). TLR7 is important for the generation of ABCs (64–69). Selective deletion of ABCs in the MRL.Fas*^lpr^* model improves nephritis and reduces T cell activation (70). Recently, Luo et al. have shown that expression of a conditional *Ncf1*-knockin allele in CD11c-expressing cells of NOX2-deficient mice alleviates lupus in both the pristane-induced lupus and spontaneous *Yaa* models. The authors attribute this phenotype to a regulatory function of NOX2 in pDCs (27). In light of our finding that NOX2 is an important regulator in B cells, and that CD11c is also expressed on ABCs, restoration of NOX2 in ABCs may well have contributed to the reduced disease burden observed by Luo et al.

It is perhaps expected that NOX2 in myeloid cells would regulate the pathogenesis of SLE, since the expression and function of NOX2 in macrophages, DCs, and neutrophils is well documented (7–10, 27–30). Indeed, others have shown macrophage activation in NOX2-deficient animals subjected to collagen-induced arthritis (71). As noted, pDCs have also been implicated in pristane-induced SLE (27). Cell-selective restoration of NOX2 function in the context of global deletion ameliorated disease in both models, establishing the importance of the target cells. Here, we took a complementary approach, using cell-selective deletion. As with both approaches, the limited efficiency and specificity of existing myeloid Cre strains in turn limits the ability to directly assess the differential contribution of myeloid subsets. In our case, we found an effect using *LysM-Cre*, which efficiently targets neutrophils but variably and partially targets macrophage populations (53). *Cybb* deficiency in the myeloid compartment, mediated by *LysM-Cre* in both our chimeric and conditional KO systems, does not fully recapitulate the *Cybb* global KO or total hematopoietic chimera phenotypes. The lack of a robust phenotype could be, in part, due to incomplete deletion of the allele. However, our combined results establish that NOX2 fulfills distinct, nonredundant regulatory functions across multiple cell types, which could also explain the partial phenotypes seen with macrophage/monocyte- and neutrophil-specific deletion.

NOX2-dependent ROS are critical for the formation of classical neutrophil extracellular traps (NETs) (72, 73). NETs were postulated to be a major source of autoantigen and were thought to consequently drive disease in SLE (74–83). However, others and we have shown that abrogating NET formation by genetically deleting or pharmacologically inhibiting NOX2 (19–27), peptidyl arginine deiminase, type IV (23, 84), and neutrophil elastase (85) exacerbated rather than ameliorated SLE phenotypes, or at best, had no effect. The data that we present here showing that conditional deletion of *Cybb* in neutrophils using *MRP8 Cre* did not impact SLE, along with that of the Homdahl group (27), strongly argues against the hypothesis that NET-derived autoantigens drive SLE. Rather, exacerbated SLE observed in the absence of NETs suggests that NETs may be immunomodulatory in SLE. Supporting a negative regulatory role for NETs, NOX2-dependent NETs can form aggregated structures that degrade proinflammatory cytokines (28). As targeting of *Cybb* in neutrophils had no effect on disease, it is unlikely that worse SLE observed in NOX2 deficiency can be explained by the absence of immunoregulatory NETs.

An important component of our work is that we identify a mechanism by which NOX2 curtails autoimmunity — NOX2 reduces inflammation by selectively regulating TLR7. It is well established that TLR7 promotes SLE, whereas TLR9 suppresses systemic autoimmunity (41, 51, 61, 66, 86–95). TLR7 is a major driver of autoantibody responses to RNA-associated autoantigens in multiple models of SLE (41, 61, 86, 88, 96–99). NOX2-deficient SLE-prone mice have increased anti-RNA and anti-Sm titers, both TLR7-dependent autoantibodies (61, 88), suggesting a stronger influence of TLR7 in NOX2-deficient mice (20). This observation led us to speculate that NOX2 may prevent autoimmunity through the regulation of the TLR7 pathway in B cells, a hypothesis supported by our findings that TLR7, but not TLR9, agonists resulted in more robust NF-κB signaling in NOX2-deficient cultured B cells. We validated these mechanistic studies with in vivo genetic experiments using meaningful disease endpoints. Global and B cell–intrinsic TLR7 deficiency rescues severe lupus in global *Cybb*-KO MRL.Fas*^lpr^* SLE-prone mice. These data align with recent work by our group showing that B cell–intrinsic TLR7 drives SLE, especially in the context of TLR9 deficiency (41).

Recently, Liu et al. speculated that NCF1 deletion promotes increased TLR signaling via dysregulation of endolysosomal trafficking in B cells (59), based on in vitro studies. This mechanism is seemingly at odds with the selective regulation of TLR7 that we observe here. They reported that NADPH oxidase deficiency led to reduced noncanonical autophagy, characterized by diminished LC3 recruitment to the endosome and delayed lysosomal fusion, a process dependent on LC3 lipidation, and known as LC3-associated phagocytosis, or LAP (100). This in turn culminates in the reduced degradation of the TLR ligand CpG and sustained TLR9 signaling (59). These in vitro studies, however, were not linked in a causal way to the humoral activation and elevated autoantibody titers in their in vivo systems. Moreover, this interpretation hinges on a conceptual framework that was based on publications claiming that NOX2 (and RUN and cysteine-rich domain–containing Beclin 1 interacting protein [RUBICON]) are essential for LAP (101, 102); however, the original papers making these claims have been retracted. Indeed, our lab along with Anne Davidson’s had already demonstrated that LC3 lipidation occurs normally without NOX2 or RUBICON (49), unlinking NOX2 from LC3-associated processes such as those discussed by Liu et al.

The model proposed by Liu et al. does not explain why TLR7 drives and TLR9 protects from SLE — in fact, it is incompatible. There is an emerging body of literature to suggest that TLR7 and TLR9 are differentially regulated in the endosomal compartment (103–105). Recently, the late endosomal biogenesis of lysosomal organelles complex 1–related complex (BORC) and GTPase adenosine 5-diphosphate ribosylation factor-like 8B (Arl8b) have been found to control intracellular TLR7, but not TLR9, turnover (104). It remains possible that NOX2 is also involved in this process.

In fact, how NOX2 selectively regulates TLR7 remains undetermined. One possible mechanism could be that NOX2-dependent ROS oxidizes essential cysteine residues in the TLR7 ectodomain; TLR9 lacks such residues (57, 58). Supporting the significance of this observation, activation of NOX2 in mice undergoing infection with influenza, a pathogen known to activate TLR7, suppressed proinflammatory cytokine production and reduced antibody production (57). Consequently, we speculate that NOX2-derived ROS in B cells protects from systemic autoimmunity by inhibiting TLR7 signaling by oxidation of Cys98. This proposed mechanism would explain the selective impact of NOX2 on TLR7 and aligns with the opposing functions of TLR7 and TLR9 in SLE pathogenesis. Moreover, it is well established that environmental factors play a critical role in triggering the onset of autoimmune diseases (106). It is intriguing to speculate that the dysregulation of NOX2-dependent cysteine oxidation may act as an environmental catalyst that precipitates the onset of disease in genetically susceptible individuals.

## Methods

Further information can be found in Supplemental Methods.

### Sex as a biological variable.

Our study examined male and female animals. Sex-dimorphic effects are reported.

### Mice.

MRL-MpJ-*Fas*^lpr^/J (MRL.Fas*^lpr^*) mice were purchased from The Jackson Laboratory (JAX; stock 000485). *Cybb^–/–^* (JAX stock 002365) (20), *LysM-Cre* (JAX stock 004781) (51, 52), *Mrp8-Cre* (JAX stock 021614) (50, 51), *CD19-Cre* (JAX stock 006785) (55, 56), *Rosa26-eGFP-DTA* (JAX stock 032087) (107, 108), and *Tlr7*-deficient (61) C57BL/6 strains were backcrossed with MRL.Fas*^lpr^* mice as previously described for at least 9 generations. A *Tlr7* conditional–KO allele was previously generated directly on the MRL.Fas*^lpr^* background using in vitro fertilization and CRISPR/Cas9 technology (41). *CD4-Cre* C57BL/6 (JAX stock 022071) (54) mice were backcrossed with the *Fas*-sufficient MRL/MpJ (JAX stock 00486) strain for at least 10 generations. *CD4-Cre* MRL/MpJ mice were bred with MRL.Fas*^lpr^* *Fas*-deficient lines to generate *CD4-Cre^–/+^* MRL.Fas*^lpr^* mice.

*Cybb^fl/fl^* MRL.Fas*^lpr^* mice were generated by in vitro fertilization and CRISPR/Cas9 technology, as previously described (49, 109). Two *loxP* sites were inserted in introns 3 and 4 flanking exon 4 of *Cybb*. To facilitate screening of founder mice and subsequent genotyping, an EcoR1 restriction site was added adjacent to each *loxP* site.

*LysM-Cre*, *MRP8-Cre*, *CD4-Cre*, or *CD19-Cre* MRL.Fas*^lpr^* mice were crossed with *Cybb^fl/fl^* MRL.Fas*^lpr^* mice. To generate mice for experimental cohorts, we intercrossed *Cybb^fl/fl^* *Cre^+/–^* with *Cybb^fl/fl^* MRL.Fas*^lpr^* mice. SLE pathology was assessed at 18–20 weeks of age in the *Cybb^fl/fl^ LysM-Cre* and *Cybb^fl/fl^ Mrp8-Cre* cohorts. *Cybb^fl/fl^ CD4-Cre* males and females were euthanized at 22 weeks and 19 weeks of age, respectively. Disease was evaluated at 16–17 weeks of age in *Cybb^fl/fl^ CD19-Cre* cohorts.

To generate the *Tlr7*-KO *Cybb*-KO cohort, *Tlr7*-deficient and *Cybb*-deficient mice were intercrossed until both alleles were homozygous. Because both *Cybb* and *Tlr7* are on the X chromosome, crosses to obtain control and experimental female mice were done in parallel; control and experimental male mice were littermates. Disease was evaluated at 15–16 weeks of age.

To generate the *Tlr7^fl/Y^* *CD19-Cre^+/–^* (male) and *Tlr7^fl/fl^* *CD19-Cre^+/–^* (female) cohorts, we crossed the TLR7 conditional KO allele with *CD19-Cre* MRL.Fas*^lpr^*. The experimental cohort was generated by intercrossing *Tlr7^fl/fl^* *CD19-Cre^+/–^* or *Tlr7^fl/Y^* CD19 Cre*^+/–^* mice with *Cybb^–/Y^* or *Cybb^–/–^* MRL.Fas*^lpr^* mice until both the *Tlr7*-floxed and *Cybb* alleles were homozygous. Disease parameters were evaluated at 15–16 weeks of age.

*Cybb*-deficient (B6.129S-*Cybb^tm1Din^*/J; JAX stock 002365) (110) and WT (C57BL/6; JAX stock 000664) mice were aged to indicated time points prior to the in vitro experiments.

### BM chimeras.

To generate total reciprocal BM chimeras, *Cybb^–/–^* or *Cybb^+/+^* MRL.Fas*^lpr^* mice were irradiated and reconstituted with either *Cybb*-sufficient or *Cybb*-deficient MRL.Fas*^lpr^* BM. Mixed-ratio BM chimeras were generated by reconstituting irradiated WT MRL.Fas*^lpr^* recipients with WT or *Cybb^–/–^* MRL.Fas*^lpr^* BM at a ratio of 80:20. Cohorts were aged for 12 or 16–18 weeks after irradiation as indicated and SLE pathology was assessed.

To delete *Cybb* selectively in the myeloid compartment, we used a mixed BM chimera strategy (Figure 4A). In brief, we crossed the *LysM-Cre* with the *Rosa26-eGFP-DTA* strains on the MRL.Fas*^lpr^* background (Δ*LysM*). Five- to 7-week-old MRL.Fas*^lpr^* recipients were irradiated and reconstituted with a 80:20 mixture of ΔLysM and *Cybb*-deficient (Δ*LysM Cybb^–/–^*) or *Cybb*-sufficient (Δ*LysM Cybb^+/+^*) BM. Cohorts were analyzed 16 weeks after irradiation as indicated.

All chimera, *Cybb^fl/fl^ LysM-Cre*, and *Cybb^fl/fl^ MRP8-Cre* cohorts were treated prophylactically with trimethoprim and sulfadiazine diet to prevent occult infection.

### Evaluation of SLE pathology.

MRL.Fas*^lpr^* were analyzed as previously described (20, 51, 111).

### Phosphoflow and nuclear translocation assays.

To assess NF-κB signaling downstream of TLR stimulation in B cells from *Cybb*-deficient mice, B cells were isolated and cells were rested for 1 hour at 37°C before performing the signaling assays. For the phosphoflow assay, samples were then stimulated with specific concentrations of CL097 and/or anti-IgM for the indicated time points. Cells were fixed/permeabilized (BD Cytofix/Cytoperm) and stained as indicated. Data were collected using an LSRII (BD) with FACS DIVA and analyzed using FlowJo software (BD).

For the nuclear translocation assay, relevant groups were pretreated for 1 hour with 1000 U/mL catalase (Thermo Fisher Scientific) or 100 μM hydrogen peroxide (Thermo Fisher Scientific) and then stimulated with the indicated concentrations of CL097, CpG ODN 1826, and/or anti-IgM for 45 minutes. Cells were then fixed followed by permeabilization and staining. Samples were run on an Imagestream^X^ Mk II imaging flow cytometer (Amnis) and analyzed using the IDEAS software (Amnis).

Antibodies used for B cell isolation via negative selection were as follows: anti-CD4–biotin (in-house conjugated, clone GK1.5), anti-CD8–biotin (in-house conjugated, clone TIB-105), anti-CD43–biotin (in-house conjugated; clone S7), anti-CD138–biotin (in-house conjugated, clone 281-2), and anti-GR1–biotin (BioLegend, clone RB6-8C5).

### Statistics.

Statistical analysis was performed using Prism 9.0 (GraphPad). A log-rank test was used to determine statistical significance between Kaplan-Meier curves. A 2-tailed or 1-tailed Mann-Whitney *U* test, 2-tailed Student’s *t* test, 2-way ANOVA with Holm-Šidák correction, Kruskal-Wallis test with post hoc Dunn’s test, or a Fisher’s exact test was performed where indicated and appropriate. A *P* value of less than 0.05 was considered statistically significant.

### Study approval.

Animal studies were approved by the University of Pittsburgh Institutional Animal Care and Use Committee.

### Data availability.

The data underlying Figures 1–7, Supplemental Figures 1–3, and Supplemental Tables 1–2 are available in the published article, its online supplemental material, and the Supporting Data Values file.

## Author contributions

RAG, HAC, AM, KMN, JST, and AMC performed experiments and analyzed data. SIB and MK performed histopathological evaluation of the kidneys. SG and RAG designed the *Cybb-*floxed allele. SG and JST designed the *Tlr7*-floxed allele. AP generated the *CD4-Cre* mice on the MRL background. RAG, HAC, and MJS designed experiments and wrote the manuscript. The order of co–first authors was determined by the length of time each author contributed to this study.

## Supplementary Material

Supplemental data

Supporting data values

## Version 1 07/23/2024

In-Press Preview

## Version 2 08/22/2024

Electronic publication
