# Supplementary material for: NADPH oxidase in B cells and macrophages protects against murine lupus by regulation of TLR7
Source: JCI Insight. 2024 Jul 23;9(16):e178563. doi: 10.1172/jci.insight.178563 (PMC11343599; doi:10.1172/jci.insight.178563)
Supplement: Supplemental data [file jciinsight-9-178563-s083.pdf]

Supplemental Materials

Supplemental Table 1: Cell composition by FACS or number of antibody forming cells (AFCs) by ELISPOT in MRL.Fas^{lpr} spleens from indicated strains.

	**Total *Cybb*$^{-/-}$ chimeras**							
**Cell Type (FACS)**	**Donor genotype**							
**Percent ± SEM**	**Wild-type (Male)**	**n**	***Cybb*$^{-/y}$ (Male)**	**n**	**Wild-type (Female)**	**n**	***Cybb*$^{-/-}$ (Female)**	**n**
Neutrophils	1.8±0.7	40	2.9±0.3	33	3.8±0.4	26	5.2±0.5	24
Macrophages	4.2±0.4	40	5.7±0.6	33	5.7±0.4	26	7.3±0.6	24
Conventional DCs	0.5±0.04	40	0.62±0.05	33	0.45±0.03	26	0.8±0.1	24
Plasmacytoid DCs	0.4±0.02	40	0.30±0.02	33	0.27±0.02	26	0.3±0.02	24
B cells	35.9±1.10	40	35.1±1.6	33	25.0±1.3	25	25.5±1.4	24
Plasmablasts	2.1±0.2	40	2.5±0.2	33	3.4±0.5	25	2.9±0.5	24
AFCs	1.7±0.1	40	2.2±0.2	33	2.5±0.4	25	2.5±0.4	24
Total T cells	37.3±1.5	40	33.4±1.8	33	43.2±1.3	26	42.7±1.7	24
CD4 T cells	19.5±0.8	40	18.1±0.9	33	20.4±0.09	26	19.6±0.9	24
Activated CD4 T cells (% of CD4)	64.3±2.2	40	61.9±2.1	33	59.7±3.2	26	59.4±2.9	24
CD8 T cells	7.7±0.4	40	6.4±0.5	33	8.1±0.5	26	6.9±0.5	24
Activated CD8 T cells (% of CD8)	25.8±1.2	40	25.7±1.6	33	27.9±1.3	26	26.7±1.4	24
	**Δ*LysM Cybb*$^{-/-}$ chimeras**							
**Cell Type (FACS)**	***D*onor genotype**							
**Percent ± SEM**	**Control (Male)**	**n**	**Δ*LysM Cybb*$^{y/-}$ (Male)**	**n**	**Control (Female)**	**n**	**Δ*LysM Cybb*$^{-/-}$ (Female)**	**n**
Neutrophils	2.0±0.2	19	2.2±0.2	20	4.5±1.0	8	5.0±0.8	8
Macrophages	4.8±0.9	19	5.3±0.8	20	6.5±0.5	8	8.2±0.6	8
Conventional DCs	0.6±0.07	19	0.6±0.05	20	0.6±0.06	8	0.9±0.1	8
Plasmacytoid DCs	0.3±0.02	19	0.3±0.02	20	0.3±0.04	8	0.3±0.02	8
B cells	32.4±1.6	19	32.2±1.35	20	20.7±2.1	8	25.0±2.2	8
Plasmablasts	1.8±0.2	19	2.2±0.3	20	0.5±0.08	8	0.8±0.1	8
AFCs	1.5±0.2	19	1.8±0.2	20	0.4±0.07	8	0.7±0.1	8
Total T cells	32.4±2.5	19	33.6±2.3	20	42.9±3.1	8	44.8±1.6	8
CD4 T cells	19.2±1.4	19	19.9±1.3	20	21.8±1.9	8	20.9±0.8	8

Activated CD4 T cells (% of CD4)	67.0±2.2	19	64.9±2.6	20	50.3±2.1	8	51.9±0.8	8
CD8 T cells	6.3±0.6	19	6.1±0.5	20	7.8±0.4	8	6.9±0.6	8
Activated CD8 T cells (% of CD8)	32.3±1.1	19	31.5±1.6	20	30.9±2.5	8	30.7±2.3	8
**ELISPOT (# AFCs per spleen)**								
Igκ	5.8x10^{5}±7.4x10^{4}	19	1.1x10^{6}±1.2x10^{5}	20	1.2x10^{6}±3.4x10^{5}	8	1.3x10^{6}±2.8x10^{5}	8
IgG1	5.1x10^{5}±9.3x10^{4}	19	1.1x10^{6}±2.7x10^{5}	18	7.0x10^{5}±2.9x10^{5}	8	7.4x10^{5}±2.9x10^{5}	8
IgG2A	3.4x10^{5}±5.6x10^{4}	19	4.4x10^{5}±6.7x10^{4}	20	3.2x10^{5}±8.7x10^{4}	8	6.0x10^{5}±1.8x10^{5}	8
IgM	3.5x10^{5}±7.8x10^{4}	19	3.2x10^{5}±5.2x10^{4}	20	2.9x10^{5}±4.0x10^{4}	8	3.2x10^{5}±7.1x10^{4}	8
	***Cybb*$^{fl/fl}$ *LysM*-Cre**							
**Cell Type (FACS)**	**Genotype**							
**Percent ± SEM**	***Cybb*$^{fl/y}$ (Male)**	**n**	***Cybb*$^{fl/y}$ *LysM-Cre*$^{+/-}$ (Male)**	**n**	***Cybb*$^{fl/fl}$ (Female)**	**n**	***Cybb*$^{fl/fl}$*LysM-Cre*$^{+/-}$ (Female)**	**n**
Neutrophils	1.7±0.2	24	1.6±0.2	21	1.0±0.2	13	1.6±0.3	16
Macrophages	1.9±0.2	24	2.2±0.2	21	1.4±0.2	13	1.7±0.2	16
Conventional DCs	0.2±0.02	24	0.3±0.02	21	0.2±0.03	13	0.2±0.02	16
Plasmacytoid DCs	0.03±0.00	24	0.03±0.00	21	0.02±0.00	13	0.02±0.00	16
B cells	25.4±1.7	24	31.5±2.2	21	18.4±1.7	13	19.0±2.7	16
Plasmablasts	5.3±0.7	24	5.5±0.6	21	9.6±0.7	13	10.8±2.0	16
AFCs	1.4±0.2	24	1.8±0.2	21	3.0±0.4	13	2.1±0.2	16
Total T cells	28.6±1.8	24	29.5±1.3	21	28.3±1.6	13	35.8±4.4	16
CD4 T cells	7.1±0.4	24	7.1±0.5	21	6.9±0.50	13	7.2±0.8	16
Activated CD4 T cells (% of CD4)	78.9±0.7	24	78.6±1.4	21	79.8±1.9	13	75.2±1.4	16
CD8 T cells	8.0±0.7	24	8.6±0.5	21	6.9±0.5	13	7.2±1.2	16
Activated CD8 T cells (% of CD8)	51.5±1.7	24	54.8±2.4	21	59.0±2.4	13	54.6±1.6	16
	***Cybb*$^{fl/fl}$ *MRP8*-Cre**							
**Cell Type (FACS)**	**Genotype**							
**Percent ± SEM**	***Cybb*$^{fl/y}$ (Male)**	**n**	***Cybb*$^{fl/y}$*MRP8-Cre*$^{+/-}$ (Male)**	**n**	***Cybb*$^{fl/fl}$ (Female)**	**n**	***Cybb*$^{fl/fl}$*MRP8-Cre*$^{+/-}$ (Female)**	**n**
Neutrophils	4.7±0.4	12	3.8±0.4	8	5.0±1.1	6	5.1±0.6	5
Macrophages	3.6±0.3	12	3.8±0.4	8	3.0±0.3	6	2.7±0.3	5
Conventional DCs	0.3±0.03	12	0.3±0.06	8	0.2±0.06	6	0.4±0.07	6

Plasmacytoid DCs	0.1±0.02	12	0.1±0.02	8	0.09±0.03	6	0.1±0.02	6
B cells	32.1±3.4	12	32.6±2.3	8	17.7±1.4	6	18.4±3.5	6
Plasmablasts	1.5±0.3	12	1.9±0.4	8	2.2±0.3	6	2.2±0.3	6
AFCs	1.0±0.2	12	1.3±0.4	8	1.1±0.23	6	1.0±0.3	6
Total T cells	48.5±3.7	12	49.7±3.0	8	61.9±2.4	6	60.5±3.3	6
CD4 T cells	13.4±1.0	12	13.3±0.7	8	13.0±0.6	6	12.4±0.7	6
Activated CD4 T cells (% of CD4)	72.9±1.1	12	70.1±1.5	8	68.5±2.6	6	69.8±2.7	6
CD8 T cells	14.2±1.2	12	13.6±1.3	8	14.1±1.2	6	13.4±1.0	6
Activated CD8 T cells (% of CD8)	33.2±2.0	12	35.5±2.4	8	34.3 ±3.1	6	36.5±1.8	6
	***Cybb$^{fl/fl}$ CD4*-Cre**							
**Cell Type (FACS)**	**Genotype**							
**Percent ± SEM**	***Cybb$^{fl/y}$* (Male)**	**n**	***Cybb$^{fl/y}$CD4-Cre$^{+/-}$* (Male)**	**n**	***Cybb$^{fl/fl}$* (Female)**	**n**	***Cybb$^{fl/fl}$CD4-Cre$^{+/-}$* (Female)**	**n**
Neutrophils	10.4±1.1	19	10.7±2.9	10	33.7±6.7	9	32.3±4.1	19
Macrophages	8.3±1.0	19	7.9±1.5	10	7.8±1.6	9	9.3±1.2	19
Conventional DCs	0.6±0.04	20	0.7±0.07	10	0.42±0.05	9	0.6±0.04	19
Plasmacytoid DCs	0.2±0.03	8	0.2±0.04	3	0.2±0.02	9	0.2±0.02	19
B cells	16.9±1.8	19	20.8±2.6	10	10.7±1.6	9	12.7±1.2	19
Plasmablasts	0.6±0.04	19	0.6±0.09	10	1.0±0.3	9	1.4±0.2	19
Total T cells	50.4±2.9	20	43.1±6.1	10	50.76±4.7	9	49.9±3.3	19
CD4 T cells	24.7±2.0	19	26.9±3.9	10	21.3±1.6	9	22.9±1.5	19
Activated CD4 T cells (% of CD4)	74.1±2.1	19	75.2±2.0	10	69.5±1.5	9	72.0±1.6	19
CD8 T cells	17.9±1.0	19	21.7±1.7	10	16.4±1.9	9	17.3±1.0	19
Activated CD8 T cells (% of CD8)	16.6±1.9	19	20.0±4.0	10	19.4±2.8	9	18.4±1.9	19
	***Cybb$^{fl/fl}$ CD19*-Cre**							
**Cell Type (FACS)**	**Genotype**							
**Percent ± SEM**	***Cybb$^{fl/y}$* (Male)**	**n**	***Cybb$^{fl/y}$CD19-Cre$^{+/-}$* (Male)**	**n**	***Cybb$^{fl/fl}$* (Female)**	**n**	***Cybb$^{fl/fl}$CD19-Cre$^{+/-}$* (Female)**	**n**
Neutrophils	4.0±0.4	17	5.1±0.5	19	4.0±0.7	11	5.7±0.8	11
Macrophages	0.4±0.03	17	0.5±0.06	19	0.3±0.02	11	0.4±0.03	11
Conventional DCs	0.4±0.03	17	0.3±0.02	19	0.4±0.02	11	0.5±0.05	11
Plasmacytoid DCs	0.2±0.02	17	0.2±0.02	19	0.2±0.01	11	0.2±0.02	11
B cells	27.9±2.2	17	22.9±2.1	19	18.8±2.0	11	18.3±3.5	11

Plasmablasts	3.4±0.4	17	3.3±0.4	19	4.7±0.7	11	4.0±0.8	11
Age-associated B cells	0.7±0.07	17	0.6±0.1	19	0.6±0.1	11	0.6±0.1	11
Germinal center B cells	0.3±0.05	17	0.4±0.1	19	0.3±0.06	11	0.4±0.1	11
Follicular B cells	14.0±1.0	17	12.0±1.4	19	11.9±1.8	11	9.8±1.5	11
Marginal zone B cells	11.8±1.1	17	10.3±1.2	19	6.8±0.07	11	8.0±2.0	11
Non-DN total T cells	26.2±1.1	17	24.8±1.0	19	23.7±2.0	11	23.1±1.1	11
CD4 T cells (% of non-DN T cells)	46.3±1.1	17	50.1±2.5	19	54.7±2.2	11	50.5±2.6	11
Activated CD4 T cells (% of CD4)	68.2±2.6	17	70.4±2.8	19	71.5±2.8	11	66.8±3.9	11
CD8 T cells (% of non-DN T cells)	50.2±1.0	17	44.9±2.4	19	41.4±2.3	11	43.8±2.4	11
Activated CD8 T cells (% of CD8)	21.8±2.6	17	26.4±2.3	19	26.2±2.9	11	23.3±3.1	11

	Tlr7$^{-/-}$* and *Cybb$^{-/-}$							
**Cell Type (FACS)**	**Genotype**							
**Percent ± SEM**	***Tlr7$^{+/y}$ Cybb$^{-/y}$* (Male)**	**n**	***Tlr7$^{-/y}$ Cybb$^{-/y}$* (Male)**	**n**	***Tlr7$^{+/+}$ Cybb$^{-/-}$* (Female)**	**n**	***Tlr7$^{-/-}$ Cybb$^{-/-}$* (Female)**	**n**
Neutrophils	3.6±0.5	13	3.2±0.3	17	5.2±03	14	3.1±0.7	9
Macrophages	2.5±0.4	13	1.2±0.2	17	0.3±0.03	14	0.3±0.03	9
Conventional DCs	0.3±0.03	13	0.3±0.02	17	0.5±0.02	16	0.4±0.05	11
Plasmacytoid DCs	0.3±0.03	13	0.4±0.02	17	0.2±0.01	16	0.3±0.04	11
B cells	27.9±1.6	13	36.7±1.4	17	14.6±1.4	16	31.9±1.4	11
Plasmablasts	1.1±0.2	13	1.0±0.2	17	2.0±0.23	16	1.4±0.2	11
Age-associated B cells	1.1±0.2	13	1.3±0.2	17	0.4±0.03	14	1.0±0.1	9
Follicular B cells	24.1±4.2	13	40.7±2.4	17	32.0±1.9	16	45.1±2.5	11
Marginal zone B cells	37.1±2.4	13	37.7±1.8	17	31.3±2.1	16	32.8±2.0	11
CD4 T cells (% of T cells)	38.3±2.0	13	36.0±1.8	17	17.5±2.1	16	24.1±4.0	11
Activated CD4 T cells (% of CD4)	86.7±2.1	13	80.7±2.4	17	82.6±1.2	16	78.1±3.5	11
Naive CD4 T cells (% of CD4)	7.12±1.8	13	11.4±1.4	17	6.3±1.0	16	6.3±1.0	11

CD8 T cells (% of T cells)	28.5±1.5	13	32.0±1.8	17	15.9±1.6	16	28.5±3.0	11
Activated CD8 T cells (% of CD8)	66.1±1.5	13	77.0±0.9	17	72.5±1.3	16	71.2±1.0	11
Naive CD8 T cells (% of CD8)	16.2±2.0	13	15.9±1.0	17	18.2±1.2	16	17.78±1.3	11

	Cybb$^{-/-}$ Tlr7$^{fl/fl}$ CD19-Cre							
**Cell Type (FACS)**	**Genotype**							
**Percent ± SEM**	***Cybb$^{-/y}$ Tlr7$^{fl/y}$* (Male)**	**n**	***Cybb$^{-/y}$ Tlr7$^{fl/y}$ CD19-Cre$^{+/-}$* (Male)**	**n**	***Cybb$^{-/-}$ Tlr7$^{fl/fl}$* (Female)**	**n**	***Cybb$^{-/-}$ Tlr7$^{fl/fl}$ CD19-Cre$^{+/-}$* (Female)**	**n**
Neutrophils	4.49±0.38	20	4.62±0.22	14	7.24±1.29	10	5.92±0.30	12
Macrophages	0.71±0.08	20	0.73±0.09	14	0.51±0.06	10	0.51±0.06	12
Conventional DCs	0.76±0.04	20	0.96±0.05	14	0.61±0.13	12	1.12±0.09	14
Plasmacytoid DCs	0.38±0.02	20	0.52±0.03	14	0.27±0.04	12	0.41±0.03	14
B cells	19.32±1.97	20	20.70±1.31	14	15.88±1.97	12	16.85±1.30	14
Plasmablasts	2.09±0.23	20	2.15±0.22	14	2.69±0.46	12	2.21±0.31	14
Age-associated B cells	1.88±0.21	20	1.95±0.19	14	2.49±0.31	10	2.56±0.27	12
Follicular B cells (% of B cells)	35.03±2.44	20	39.79±2.38	14	28.43±2.58	12	32.20±2.50	14
Marginal zone B cells (% of B cells)	27.78±1.92	20	31.84±1.56	14	26.13±2.79	12	30.89±1.57	14
CD4 T cells (% of T cells)	30.91±1.29	20	30.77±1.38	14	31.43±2.43	12	33.61±1.92	14
Activated CD4 T cells (% of CD4)	79.40±1.31	20	80.94±1.04	14	83.73±1.36	12	82.17±1.32	14
Naive CD4 T cells (% of CD4)	6.9±0.9	20	6.69±0.7	14	4.1±0.7	12	5.7±0.5	14
CD8 T cells (% of T cells)	24.40±0.54	20	23.59±0.99	14	17.59±1.62	12	18.92±0.45	14
Activated CD8 T cells (% of CD8)	78.5±1.1	20	78.4±0.8	14	79.72±1.12	12	76.17±1.01	14
Naive CD8 T cells (% of CD8)	15.2±1.1	20	14.8±0.6	14	12.51±0.98	12	13.31±0.67	14

Grey comparisons indicate statistically significant differences ($p<0.05$) by Student's t test (FACS) or by Mann-Whitney U test (ELISPOT).

Supplemental Table 2: *Cybb* deletion efficiency.

Strain	Cell Type	n	Mean % deletion efficiency ± SEM
Total *Cybb*$^{-/-}$ chimeras	Neutrophils	12	99.7±0.1
	Macrophages	12	98.9±0.3
	B cells	12	99.6±0.1
	T cells	10	84.9±3.2
Δ*LysM Cybb*$^{-/-}$ chimeras	Neutrophils	12	88.1±2.1
	Macrophages	12	46.3±2.0
	B cells	12	30.7±2.4
	T cells	10	16.1±5.2
20% *Cybb*$^{y/-}$; 80% WT chimeras	Neutrophils	4	20.8±1.3
	Macrophages	5	36.3±3.8
	B cells	5	28.8±4.0
	T cells	5	14.3±2.8
Cybb$^{fl/fl}$ *LysM-Cre*$^{+/-}$ (males)	Neutrophils	19	68.3±2.2
	Macrophages	19	26.3±6.00
Cybb$^{fl/fl}$ *LysM-Cre*$^{+/-}$ (females)	Neutrophils	15	68.6±2.2
	Macrophages	15	42.9±5.9
Cybb$^{fl/fl}$ *MRP8-Cre*$^{+/-}$ (males)	Neutrophils	10	89.4±0.8
Cybb$^{fl/fl}$ *MRP8-Cre*$^{+/-}$ (females)	Neutrophils	6	88.3±0.8
Cybb$^{fl/fl}$ CD4-Cre$^{+/-}$ (males)	$CD4^+$ T cells	3	96.3±2.5
	$CD8^+$ T cells	3	87.4±7.40
Cybb$^{fl/fl}$ CD4-Cre$^{+/-}$ (females)	$CD4^+$ T cells	5	96.00±1.5
	$CD8^+$ T cells	5	74.5±6.0
Cybb$^{fl/fl}$ *CD19-Cre*$^{+/-}$	Neutrophils	5	-8.7±13.5
	Macrophages	5	37.2±6.4
	B cells	5	85.6±2.5
	T cells	5	-0.04±5.5

Supplemental Figure 1. The absence of *Cybb* in the stromal compartment does not affect nephritis or the autoantibody response in SLE prone mice.

(A-F) Reciprocal bone marrow (BM) chimeras were generated with male MRL.Fas^{lpr} donors and recipients of the indicated genotypes. **(A)** Proteinuria scores. **(B)** Glomerulonephritis scores. **(C)** Interstitial nephritis scores. **(D-F)** Anti-nucleosome **(D)**, anti-Sm **(E)**, and anti-RNA **(F)** antibody titers. Data are from mice with the indicated *Cybb* donor and recipient genotypes at 12 weeks post irradiation. Data representation and statistics are as in Figure 1. *Cybb*$^{-/y}$ (donor)→ *Cybb*$^{-/y}$ (recipient) n=8*;* Wild-type (Wt)→ *Cybb*$^{-/y}$ n=7 (panel A) and n=8 (panels B-F); *Cybb*$^{-/y}$ →Wt *n*=9 (panel A) and n=8 (panels B-F); Wt →Wt n=6.

Supplemental Figure 2. Deletion of *Cybb* in 20% of the hematopoietic compartment does not affect nephritis or the autoantibody response in SLE prone mice. (A-F) Total or mixed bone marrow (BM) chimeras were generated in wild-type (WT) male MRL.Fas^{lpr} recipients with either *Cybb*-sufficient or -deficient BM at indicated percentages. **(A)** Proteinuria scores. **(B)** Glomerulonephritis scores. **(C)** Interstitial nephritis scores. **(D-F)** Anti-nucleosome **(D)**, anti-Sm **(E)**, and anti-RNA **(F)** antibody titers. Data are from mice of the indicated *Cybb* donor *g*enotype at 16 weeks post irradiation. Donor genotype: 100% *Cybb*$^{y/-}$ n=10 (panel A) and n=11 (panels B-F); 20% *Cybb*$^{y/-}$ and 80% WT n=8 (panel A) and n=9 (panel B-F); 100% control n=12. Data representation and statistics are as in Figure 1 unless otherwise indicated. A Kruskal-Wallis test with post-hoc Dunn's test was used to determine statistical significance in panels A-F.

Supplemental Figure 3. T cell *Cybb* deficiency does not affect murine lupus. (A) Proteinuria scores. **(B)** Glomerulonephritis scores. **(C)** Interstitial nephritis scores. **(D)** Dermatitis scores. **(E)** Spleen and **(F)** axillary lymph node weights. **(G-I)** Anti-nucleosome **(G)**, anti-Sm **(H)**, and anti-

RNA **(I)** antibody titers. Data are from mice of the indicated genotypes at 19 and 22 weeks of age in female and male MRL.Fas^{lpr} mice respectively (*Cybb*$^{fl/y}$ males n=24 (panels A-F and H), n=23 (panels G and I); *Cybb*$^{fl/y}$ *CD4-Cre*$^{+/-}$ males n=13; *Cybb*$^{fl/fl}$ females n=12; *Cybb*$^{fl/fl}$ *CD4-Cre*$^{+/-}$ females n=20). Data representation and statistics are as in Figure 1.

Supplemental Methods

Sex as a biological variable

Our study examined male and female animals. Sex-dimorphic effects are reported.

Mice

MRL-MpJ-*Fas*^{lpr}/J (MRL.Fas^{lpr}) mice were purchased from the Jackson Laboratory (Catalogue# 000485). We purchased *Cybb*-deficient (Catalogue# 002365) mice from the Jackson Laboratory and backcrossed them to the MRL.Fas^{lpr} strain for at least 9 generations as previously described (1). *Tlr7*-deficient MRL.Fas^{lpr} mice were generated as previously described (2). A *Tlr7* conditional-knockout (KO) allele was generated directly on the MRL.Fas^{lpr} background as previously described using in vitro fertilization and CRISPR-Cas9 technology (3). *LysM-Cre* (Catalogue# 004781) (4, 5), *Mrp8-Cre* (Catalogue# 021614) (5, 6), *CD19-Cre* (Catalogue # 006785) (7, 8), and *Rosa26-eGFP-DTA* (Catalogue# 032087) (9, 10) C57BL/6 strains were backcrossed to MRL.Fas^{lpr} mice as previously described for at least 9 generations. CD4-Cre C57BL/6 (Catalogue# 022071) (11) mice were backcrossed to the *Fas*-sufficient MRL/MpJ (Catalogue# 00486) by Dr. Andras Perl (State University of New York Upstate Medical University) for at least 10 generations. *CD4-Cre* MRL/MpJ mice were a kind gift from Dr. Perl and we bred *CD4-Cre*$^{-/+}$ MRL *Fas*-sufficient to MRL.Fas^{lpr} *Fas*-deficient lines to generate CD4-Cre$^{-/+}$ MRL.Fas^{lpr} mice.

Cybb-flox MRL.Fas^{lpr} mice were generated by in vitro fertilization and CRISPR-Cas9 technology (12, 13). In brief, two loxP sites were inserted in introns 3 and 4 flanking exon 4 of *Cybb*. To

facilitate screening of founder mice and subsequent genotyping, an EcoR1 restriction site was added adjacent to each loxP site. MRL.Fas^{lpr} pronuclear-stage zygotes were generated by in vitro fertilization. Resulting zygotes were microinjected by the Mouse Embryo Services Core (University of Pittsburgh) with capped Cas9 mRNA (100ng/ul), single guide RNAs (sgRNAs (50ng/ul), and Ultramer oligonucleotide (0.5mM; Integrated DNA Technologies) repair templates. Injected zygotes were cultured overnight and transferred to pseudopregnant CD1 recipient females (Taconic) to obtain potential founder mice. Mice were genotyped by PCR amplification of the target locus and RFLP analysis. Amplified PCR products were subsequently cloned into a sequencing vector (NEB) and sequenced to verify correct targeting.

Genomic sequencing of the resulting founder revealed a one base pair deletion (bold and strikethrough annotation) in the 3' Cre binding arm of the intron 4 loxP site (5'-ATAACTTCGTATAGCATACATTATACGAAGT~~**T**~~AT-3'). Based on in vitro studies showing that mutations in the loxP arms can be tolerated (14), we proceeded to test the functionality of this *Cybb-flox* allele and confirmed that deletion efficiency was consistent with prior use of Cre alleles (15) by qPCR of genomic DNA from FACS sorted cells (Supplemental table 2).

Guide RNA target sequences, Ultramer repair template sequences, and genotyping primers are listed below:

Cybb intron 3 sgRNA target sequence: 5'-GGCCTCATATTACTAGTACG<u>AGG</u>-3'
Cybb intron 4 sgRNA target sequence: 5'-GATCTTGGTGATACGTATGC<u>AGG</u>-3'

Cybb intron 3 loxP Ultramer:

CTAGAAATATATTTTGGCAAAATTCAAAACTTTAGATAACAGTGAAAAATGTATTTGCA ACAAGGCCTCATATTACTAGT**ATAACTTCGTATAATGTATGCTATACGAAGTTAT***GAA TTC*ACGAGGAAAGGAAACAAATGACTTAAGCTGGCTATCTGAACTGGGAGGGATGAC TATCATCCCTTGTCATCAACCAAATG

Cybb intron 4 loxP Ultramer:

GGTAGCTTGGATGATAGCACTGCACACCGGTAAGTCTTGGAAGACATTTGAAATGCA GAGAGCGATCTTGGTGATACGTA**ATAACTTCGTATAATGTATGCTATACGAAGTTAT** *GAATTC*TGCAGGGCCATTAGGTTGGGATCGGTTGAGTTAAGGTTAAAGCATTCAAACT ATTTCCTGATGTTAGGATTCATACCTTG

Bold text highlights the LoxP site and italics denotes the EcoR1 site.

Genotyping primers

Intron 3 loxP site

Forward: 5'-ACAAGCTCTCAGTAACTTTCAGT-3'

Reverse: 5'-TGTGAACGATTTCTTTGTTCCAGA-3'

Intron 4 loxP

Forward: 5'- TCTGAACTGGGAGGGATGACT -3'

Reverse: 5'- ACATGAGGATAGACTGTATGGCA -3'

Both loxP sites

Forward: 5'-ACAAGCTCTCAGTAACTTTCAGT-3'

Reverse: 5'-ACATGAGGATAGACTGTATGGCA-3'

The founders were backcrossed to the MRL.Fas^{lpr} strain for 2 generations to breed out potential undetected off-target gene editing events.

LysM-Cre, MRP8-Cre, CD4-Cre, or *CD19-Cre* MRL.Fas^{lpr} mice were intercrossed with *Cybb*$^{fl/fl}$ MRL.Fas^{lpr} mice. *Cre* alleles were genotyped as previously described for each strain (4-8, 11). Resulting *Cybb*$^{fl/wt}$ *Cre*$^{+/-}$ MRL.Fas^{lpr} mice were then crossed with the *Cybb*$^{fl/fl}$ MRL.Fas^{lpr} strain. To generate mice for experimental cohorts, we intercrossed *Cybb*$^{fl/fl}$ *Cre*$^{+/-}$ to *Cybb*$^{fl/fl}$ MRL.Fas^{lpr} mice. This breeding strategy allowed us to use littermate controls for each group. SLE pathology was assessed at 18-20 weeks of age in the *Cybb*$^{fl/fl}$ *LysM-Cre* and *Cybb*$^{fl/fl}$ *Mrp8-Cre* cohorts. *Cybb*$^{fl/fl}$ *CD4-Cre* males and females were euthanized at 22 weeks and 19 weeks of age respectively. Disease was evaluated at 16-17 weeks of age in *Cybb*$^{fl/fl}$ *CD19-Cre* cohorts.

To generate the *Tlr7* and *Cybb* single and double KO cohorts, *Tlr7*-deficient and *Cybb*-deficient mice were intercrossed. Because both *Cybb* and *Tlr7* are on the X-chromosome, crosses to obtain control and experimental female mice were done in parallel; control and experimental male mice were littermates. Disease was evaluated at 15-16 weeks of age.

Tlr7$^{fl/y}$ *CD19-Cre*$^{+/-}$ (male) and *Tlr7*$^{fl/fl}$ *CD19-Cre*$^{+/-}$ (female) mice were generated by crossing the TLR7-conditional KO allele to *CD19-Cre* MRL.Fas^{lpr} until the conditional allele was homozygous. The experimental cohort was generated by intercrossing *Tlr7*$^{fl/fl}$ *CD19 Cre*$^{+/-}$ or *Tlr7*$^{fl/y}$ *CD19 Cre*$^{+/-}$ mice with *Cybb*$^{-/y}$ or *Cybb*$^{-/-}$ MRL.Fas^{lpr} mice until both the *Tlr7*-floxed and

Cybb alleles were homozygous. Littermates without a Cre allele were used as controls. Disease was evaluated at 15-16 weeks of age.

Cybb-deficient (B6.129S-*Cybb*^{tm1Din}/J; Catalogue #002365) (16) and wild-type C57BL/6 (Catalogue #000664) mice were obtained from Jackson Laboratories and aged to indicated time points prior to the in vitro experiments.

Bone marrow chimeras

Bone marrow cells were obtained from either *Cybb*$^{-/-}$ or wild-type MRL.Fas^{lpr} littermate control mice. Cells were incubated in ammonium-chloride-potassium buffer (ACK) buffer (Gibco) for erythroid cell lysis and resuspended in injection buffer (1 × PBS, 10 mM Hepes, 2.5% acid citrate dextrose anticoagulant, and 0.5% penicillin/streptomycin). 5-7 week old *Cybb*$^{-/-}$ or *Cybb*$^{+/+}$ MRL.Fas^{lpr} recipient mice received 750 cGy from a cesium irradiator and were reconstituted with 8X10^{6} *Cybb*$^{-/-}$ or *Cybb*$^{+/+}$cells per mouse. The *Cybb*-sufficient controls in Figure 1, were often the *ΔLysM Cybb*$^{+/+}$ MRL.Fas^{lpr} mice described below. For the 80:20 mixed ratio bone marrow chimeras, WT MRL.Fas^{lpr} recipients received 1.6X10^{6} *Cybb*$^{-/-}$ and 6.4X10^{6} *Cybb*$^{+/+}$ cells per mouse. Cohorts were aged for 12 or 16-18 weeks post irradiation as indicated and SLE pathology was assessed.

To delete *Cybb* selectively in the myeloid compartment, we crossed the *LysM-Cre* to the *Rosa26-eGFP-DTA* strains on the MRL.Fas^{lpr} background (*ΔLysM*). The *Rosa26-eGFP-DTA* locus contains the gene for the diphtheria toxin fragment A (DTA) that is preceded by a loxP-flanked STOP cassette so that expression of the toxin is restricted to those cells expressing Cre recombinase. Expression of DTA leads to the death of that cell. Bone marrow cells from *Cybb*$^{-/-}$,

Cybb$^{+/+}$, and *ΔLysM* were isolated and processed as above. 5-7 week old MRL.Fas^{lpr} recipients were irradiated and reconstituted with a 80:20 mixture of *ΔLysM* and *Cybb*-deficient (*ΔLysM cybb*$^{-/-}$) or *Cybb*-sufficient (*ΔLysM cybb*$^{+/+}$) bone marrow. Cohorts were analyzed at 16 weeks post-irradiation as indicated.

All chimera, *Cybb*$^{fl/fl}$ *LysM-Cre*, and *Cybb*$^{fl/fl}$ *MRP8-Cre* cohorts were treated prophylactically with trimethoprim and sulfadiazine diet to prevent occult infection.

Quantitative PCR to assess Cybb deletion efficiency

To determine the efficiency and specificity of *Cybb* deletion in chimera and conditional KO cohorts, cell lysates were generated from FACS sorted splenocytes (in-house lysis buffer: 50mM Tris-Base, 50mM KCl, 0.63mM EDTA, 0.22% Tween-20, 0.22% NP-40, and 1/40 proteinase K). Genomic DNA was used as a qRT-PCR template. qRT-PCR reactions were completed in triplicate using the Kappa SYBR Green QPCR kit and analyzed on a Roche Light Cycler instrument. The amount of *Cybb* in each sample was normalized to the unaffected gene, *Tlr9*. Genomic DNA of the same cell type from *Cybb*$^{fl/fl}$ mice was used as undeleted control.

Evaluation of SLE pathology

MRL.Fas^{lpr} cohorts were evaluated as previously described (1, 5, 17). Cutaneous disease was scored based on the extent of lesions on the back and dorsum of the neck. Total surface area of the lesions was scored from 0 to 5 for an affected area up to 9.1 cm^2. One additional point was given for the presence of ear (1/4 point each) and muzzle (1/2 point) dermatitis (18). Urine dipsticks (Siemens Albustix) were used to screen for proteinuria at indicated time points. Plasma was

obtained by cardiac puncture. Kidneys were removed, bisected, formalin-fixed, paraffin embedded, and H&E stained. Kidneys were scored for glomerulonephritis (GN) and interstitial nephritis (IN) by a clinical pathologist blinded to genotype. GN was scored on a scale of 1-6: (1) normal kidney; (2) mesangial expansion, mesangial hypercellularity, and patent capillary loops; (3) enlarged glomeruli with moderate endocapillary hypercellularity; (4) as in 3 but with the addition of marked endocapillary hypercellularity and loss of patency of most capillary loops; (5) few glomeruli with necrosis [karyorrhexis] or few active [cellular or fibrocellular] or organized [fibrous] crescents; (6) many active [cellular or fibrocellular] or organized [fibrous] crescents, necrosis [karyorrhexis], and/or obliteration of glomerular architecture with segmental /global sclerosis) (5). IN was scored on a scale of 1-4 in a blinded manner by a clinical pathologist. A score of 1-4 was assigned as follows: (1) minimal inflammation [lymphocytes and plasma cells] confined to the perivascular area; (2) expansion of inflammation throughout the interstitial space but maintained in a discrete area; (3) diffuse infiltrates in over 40% of high-powered fields; (4) diffuse infiltrate throughout the entire interstitial space) (5).

Autoantibody ELISAs

Serum anti-Sm, anti-nucleosome, and anti-RNA autoantibody ELISAs were performed on MRL.Fas^{lpr} cohorts as previously described (1, 19-22). Specific antibodies were detected with alkaline phosphatase-conjugated goat anti-mouse IgG (Southern Biotech [1030-04]). Y2, BWR4, or PL2-3 (in-house) monoclonal antibodies were used as standards for the anti-Sm, anti-RNA, and anti-nucleosome measurements respectively.

ELISpot assays

AFC producing κ light chain antibodies, IgG1, IgG2a, or IgM were detected by ELISpot as previously described (7). In brief, 96-well Immulon 4 HBX plates were coated overnight at 4°C with 5 mg/ml polyclonal goat-anti mouse k (Southern Biotech; 1050-01). Nonspecific binding was blocked with 1% bovine serum albumin in PBS and samples were incubated at 37°C. Alkaline phosphatase-conjugated secondary antibodies (Southern Biotech; Igκ [1050-04], IgG1 [1070-04], IgG2a [1080-04], or IgM [1020-04]) were detected with bromo-4-chloro-3-indolyl phosphate substrate (Southern Biotech).

Flow cytometry

Flow cytometry was performed as previously described (1). In brief, spleens were homogenized and red blood cells were lysed using ACK (prepared in house). Cells were resuspended in PBS with 3% calf serum and the FcR-blocking antibody 2.4G2 (in-house). Live/dead discrimination was performed using fixable viability stain Ghost Dye Violet 510 (Tonbo). Surface and intracellular staining antibodies used in this study are listed below. Cells were fixed in 1% paraformaldehyde or Cytofix/Cytoperm (BD) where appropriate. Data were acquired using a LSRII or Fortessa (BD) with FACS DIVA software and analyzed using FlowJo.

Antibodies used for FACS staining

Antibodies used for FACS surface and intracellular staining were as follows: IA/E-PE (Biolegend, M5/114.15.2), IA/E-APC-Cy7, (Biolegend, M5/114.15.2), IA/E-BV605 (Biolegend, M5/114.15.2), Bst-2-biotin (in-house conjugated, 927), CD11c-PE/Cy7 (BD Pharmingen, HL3), CD11c-PE/Cy7 (in-house conjugated, N418), CD11c-Al488 (eBioscience, N418), CD45R-

APC/Cy7 (BD Pharmingen, RA3-6B2), SiglecH-Al647 (eBioscience, eBio440c), SiglecH-Al647 (Biolegend, 551), Ly6G-Al488 (in-house conjugated, 1A8), Ly6G-biotin (in-house conjugated, 1A8), Gr1-PE/Cy7 (Biolegend, RB6-8C5), Gr1-PE (Biolegend, RB6-8C5), CD11b-APC/Cy7 (Biolegend, M1/70), CD11b-PE (Biolegend, M1/70), CD11b-biotin (in house conjugated, M1/70), CD11b-PacBlue (in-house conjugated, M1/70), F4/80-AI647 (in-house conjugated, BM8), F4/80-APC (Biolegend, BM8), CD44-Al488 (in-house conjugated, 1M7), CD44-APC-Cy7 (Biolegend, 1M7), TcRβ-APC/Cy7 (Biolegend, H57-597), TCRβ-PE/Cy7 (Biolegend, H57-597), TCRβ-PerCP/Cy5.5 (Biolegend, H57-597), CD62L-PE/Cy7 (Biolegend, Mel-14), CD8-Al647 (in-house conjugated, TIB 105), CD4-PE (Biolegend, GK1.5), CD4-PacBlue (in-house conjugated, GK1.5), CD138-PE (BD Pharmingen, 281-2), CD138-BV605 (BD Horizon, 281-2, CD19-Pacblue (in-house conjugated, 1D3.2), CD19-Al 647 (in-house conjugated, 1D3.2), CD19-BUV-395 (BD Horizon, 1D3), kappa-Pacblue (in-house conjugated, 187.1), CD21/CD35-Al 488 (in-house conjugated, 7G6), CD21/35-PerCP/Cy5.5 (Biolegend, 7E9), CD23-biotin (in-house conjugated, B3B4), CD23 PE/Cy7 (Biolegend, B3B4), CD23-PE-Cy7 (BD Pharmingen, B3B4), IgM-Pacblue (in-house conjugated, B7-6), CD93-PE (Biolegend, AA4.1), CD317-Biotin (eBioscience, eBio927), GFP-FITC (Polyclonal, Rockland), Streptavidin-APC-efluor 780 (eBioscience). Streptavidin-BUV395 (BD Horizon), Streptavidin-PE/Cy7 (BD Pharmingen), TLR7-PE (BD Pharmingen, A94B10).

Phosphoflow and nuclear translocation assays

To assess NF-κB signaling downstream of TLR stimulation in B cells from *Cybb*-deficient mice, B were isolated as follows. Spleens were processed via mechanical dissociation and red blood cells were lysed with ammonium-chloride-potassium buffer (in-house). B cells were isolated via

negative selection using biotinylated antibodies and streptavidin magnetic beads (ThermoFisher Scientific). Cells were rested in B cell media (RPMI + 10% Fetalplex (GeminiBio), 100 units/mL penicillin (Gibco), 100ug/mL streptomycin (Gibco), 2mM Glutamax (Gibco), 10mM HEPES (Corning), and 50uM 2-mercaptothanol) for 1 hour at 37°C before performing the signaling assays.

For the phosphoflow assay, samples were then stimulated with the indicated concentrations of CL097 and/or anti-IgM for the indicated time points. Cells were fixed/permeabilized (BD Cytofix/Cytoperm) and stained as indicated. Data was collected using an LSRII (BD) with FACS DIVA and analyzed using FlowJo software.

For the nuclear translocation assay, relevant groups were pretreated for 1 hour with 1000U/mL catalase (ThermoFisher Scientific) or 100uM hydrogen peroxide (ThermoFisher Scientific) and then were stimulated with the indicated concentrations of CL097, CpG ODN 1826, and/or anti-IgM for 45 minutes. Cells were then fixed with 1.5% paraformaldehyde followed by permeabilization with PBS with 5% BSA (GeminiBio), HEPES (Corning), EDTA (Fisher Reagents), and 0.1% Triton-X. Cells were stained with the indicated antibodies and DAPI. Samples were run on an ImagestreamX MkII Imaging Flow Cytometer (Amnis) and analyzed using the IDEAS software (Amnis).

Antibodies used for B cell isolation via negative selection were as follows: anti-CD4-Biotin (in-house conjugated, GK1.5), anti-CD8-Biotin (in-house conjugated, TIB-105), anti-CD43-Biotin (in-house conjugated; S7), anti-CD138-Biotin (in-house conjugated, 281-2), and anti-GR1-Biotin (Biolegend, RB6-8C5).

Antibodies used for the phosphoflow assay were as follows: anti-NFκB-phospho-p65 PE (Cell Signaling, Ser536, 93H1); anti-CD45R APC/Cy7 (BD Pharmingen, RA3-6B2), anti-TCRb PerCP/Cy5.5 (Biolegend, H57-597), anti-CD21/35 Alexa488 (in-house conjugated; 7G6), and anti-CD23 BV510 (Biolegend, B3B4).

Antibodies for the nuclear translocation assay were as follows: anti-NFκB p-65 unconjugated (Cell Signaling, D14E12), anti-rabbit IgG-Cy3 (Invitrogen, polyclonal), anti-CD21/35-Alexa488 (in-house conjugated, 7G6), anti-CD22-Biotin (in-house conjugated, Cy34.1), and SAV-PE/Cy7 (BD Pharmingen).

Statistics

Statistical analysis was performed using Prism 9.0 (Graphpad). A log-rank test was used to determine statistical significance between Kaplan Meier curves. A two-tailed or one-tailed Mann-Whitney U test, two tailed Student T test, two-way ANOVA with Holm-Sidak correction, Kruskal-Wallis test with post-hoc Dunn's test, and a Fishers Exact test were performed where indicated and appropriate. A p value of <0.05 was considered statistically significant.

Study Approval

Animal studies were approved by the University of Pittsburgh Institutional Animal Care and Use Committee.

Data Availability

The data underlying figures 1-7, supplemental figure 1-3, and supplemental tables 1-2 are available in the published article, its online supplemental material, and the Supporting Data Values file.

Supplemental References

1. Campbell AM, Kashgarian M, and Shlomchik MJ. NADPH oxidase inhibits the pathogenesis of systemic lupus erythematosus. *Sci Transl Med.* 2012;4(157):157ra41.
2. Christensen SR, Shupe J, Nickerson K, Kashgarian M, Flavell RA, and Shlomchik MJ. Toll-like receptor 7 and TLR9 dictate autoantibody specificity and have opposing inflammatory and regulatory roles in a murine model of lupus. *Immunity.* 2006;25(3):417-28.
3. Cosgrove HA, Gingras S, Kim M, Bastacky S, Tilstra JS, and Shlomchik MJ. B cell-intrinsic TLR7 expression drives severe lupus in TLR9-deficient mice. *JCI Insight.* 2023;8(16).
4. Clausen BE, Burkhardt C, Reith W, Renkawitz R, and Forster I. Conditional gene targeting in macrophages and granulocytes using LysMcre mice. *Transgenic research.* 1999;8(4):265-77.
5. Tilstra JS, John S, Gordon RA, Leibler C, Kashgarian M, Bastacky S, et al. B cell-intrinsic TLR9 expression is protective in murine lupus. *J Clin Invest.* 2020;130(6):3172-87.
6. Passegue E, Wagner EF, and Weissman IL. JunB deficiency leads to a myeloproliferative disorder arising from hematopoietic stem cells. *Cell.* 2004;119(3):431-43.
7. Teichmann LL, Schenten D, Medzhitov R, Kashgarian M, and Shlomchik MJ. Signals via the adaptor MyD88 in B cells and DCs make distinct and synergistic contributions to immune activation and tissue damage in lupus. *Immunity.* 2013;38(3):528-40.
8. Rickert RC, Roes J, and Rajewsky K. B lymphocyte-specific, Cre-mediated mutagenesis in mice. *Nucleic Acids Res.* 1997;25(6):1317-8.

9. Teichmann LL, Ols ML, Kashgarian M, Reizis B, Kaplan DH, and Shlomchik MJ. Dendritic cells in lupus are not required for activation of T and B cells but promote their expansion, resulting in tissue damage. *Immunity.* 2010;33(6):967-78.

10. Ivanova A, Signore M, Caro N, Greene ND, Copp AJ, and Martinez-Barbera JP. In vivo genetic ablation by Cre-mediated expression of diphtheria toxin fragment A. *Genesis.* 2005;43(3):129-35.

11. Lee PP, Fitzpatrick DR, Beard C, Jessup HK, Lehar S, Makar KW, et al. A critical role for Dnmt1 and DNA methylation in T cell development, function, and survival. *Immunity.* 2001;15(5):763-74.

12. Gordon RA, Giannouli C, Raparia C, Bastacky SI, Marinov A, Hawse W, et al. Rubicon promotes rather than restricts murine lupus and is not required for LC3-associated phagocytosis. *JCI Insight.* 2022;7(7).

13. Pelletier S, Gingras S, and Green DR. Mouse genome engineering via CRISPR-Cas9 for study of immune function. *Immunity.* 2015;42(1):18-27.

14. Sheren J, Langer SJ, and Leinwand LA. A randomized library approach to identifying functional lox site domains for the Cre recombinase. *Nucleic Acids Research.* 2007;35(16):5464-73.

15. Abram CL, Roberge GL, Hu Y, and Lowell CA. Comparative analysis of the efficiency and specificity of myeloid-Cre deleting strains using ROSA-EYFP reporter mice. *Journal of immunological methods.* 2014;408:89-100.

16. Pollock JD, Williams DA, Gifford MA, Li LL, Du X, Fisherman J, et al. Mouse model of X-linked chronic granulomatous disease, an inherited defect in phagocyte superoxide production. *Nat Genet.* 1995;9(2):202-9.

17. Nickerson KM, Cullen JL, Kashgarian M, and Shlomchik MJ. Exacerbated autoimmunity in the absence of TLR9 in MRL.Fas(lpr) mice depends on Ifnar1. *Journal of immunology.* 2013;190(8):3889-94.

18. Berland R, Fernandez L, Kari E, Han JH, Lomakin I, Akira S, et al. Toll-like receptor 7-dependent loss of B cell tolerance in pathogenic autoantibody knockin mice. *Immunity.* 2006;25(3):429-40.

19. Nickerson KM, Christensen SR, Shupe J, Kashgarian M, Kim D, Elkon K, et al. TLR9 regulates TLR7- and MyD88-dependent autoantibody production and disease in a murine model of lupus. *Journal of immunology.* 2010;184(4):1840-8.

20. Christensen SR, Kashgarian M, Alexopoulou L, Flavell RA, Akira S, and Shlomchik MJ. Toll-like receptor 9 controls anti-DNA autoantibody production in murine lupus. *The Journal of experimental medicine.* 2005;202(2):321-31.

21. Monestier M, and Novick KE. Specificities and genetic characteristics of nucleosome-reactive antibodies from autoimmune mice. *Molecular immunology.* 1996;33(1):89-99.

22. Blanco F, Kalsi J, and Isenberg DA. Analysis of antibodies to RNA in patients with systemic lupus erythematosus and other autoimmune rheumatic diseases. *Clinical and experimental immunology.* 1991;86(1):66-70.

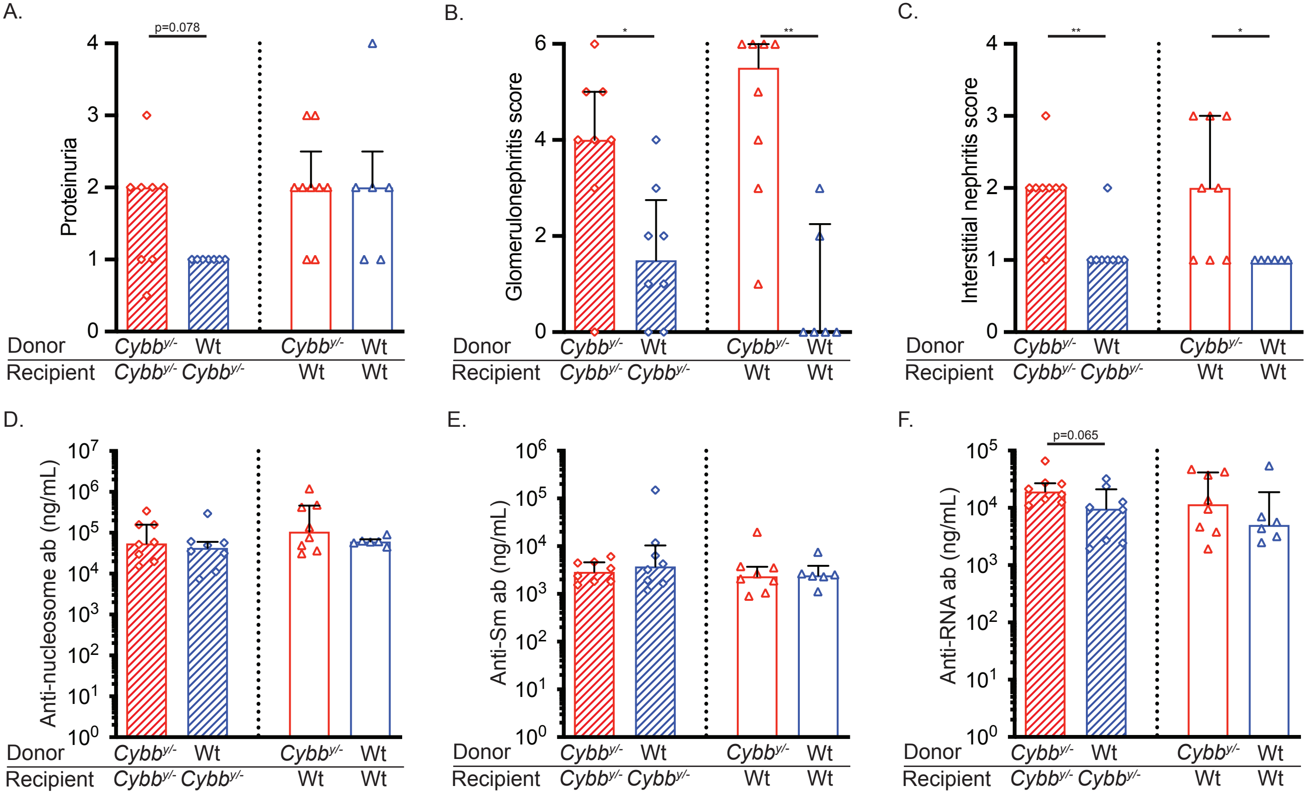

A.
p=0.078
Proteinuria
0
1
2
3
4
Donor Cybbʸ/⁻ Wt Cybbʸ/⁻ Wt
Recipient Cybbʸ/⁻ Cybbʸ/⁻ Wt Wt
B.
*
**
Glomerulonephritis score
0
2
4
6
Donor Cybbʸ/⁻ Wt Cybbʸ/⁻ Wt
Recipient Cybbʸ/⁻ Cybbʸ/⁻ Wt Wt
C.
**
*
Interstitial nephritis score
0
1
2
3
4
Donor Cybbʸ/⁻ Wt Cybbʸ/⁻ Wt
Recipient Cybbʸ/⁻ Cybbʸ/⁻ Wt Wt
D.
Anti-nucleosome ab (ng/mL)
10^0
10^1
10^2
10^3
10^4
10^5
10^6
10^7
Donor Cybbʸ/⁻ Wt Cybbʸ/⁻ Wt
Recipient Cybbʸ/⁻ Cybbʸ/⁻ Wt Wt
E.
Anti-Sm ab (ng/mL)
10^0
10^1
10^2
10^3
10^4
10^5
10^6
Donor Cybbʸ/⁻ Wt Cybbʸ/⁻ Wt
Recipient Cybbʸ/⁻ Cybbʸ/⁻ Wt Wt
F.
p=0.065
Anti-RNA ab (ng/mL)
10^0
10^1
10^2
10^3
10^4
10^5
Donor Cybbʸ/⁻ Wt Cybbʸ/⁻ Wt
Recipient Cybbʸ/⁻ Cybbʸ/⁻ Wt Wt

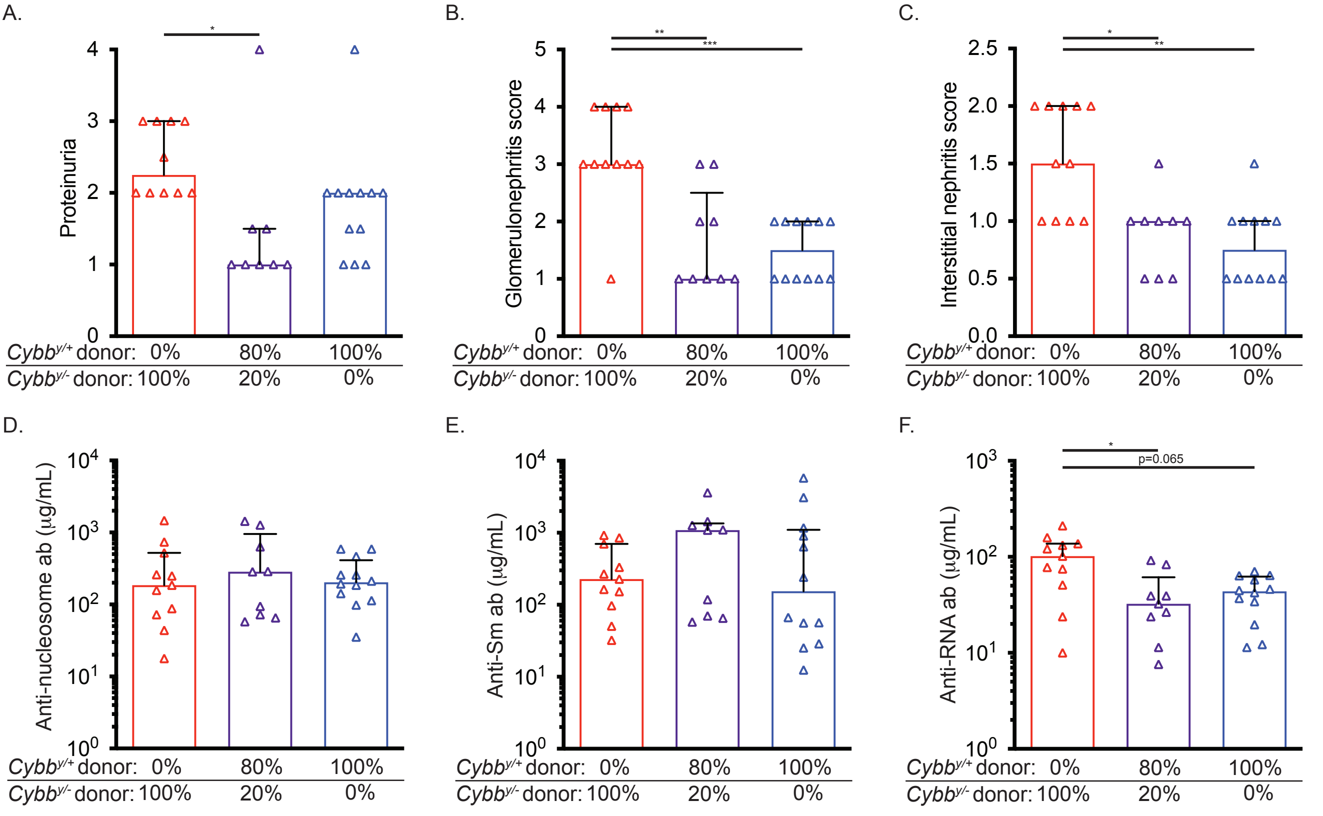

A.
Proteinuria
B.
Glomerulonephritis score
C.
Interstitial nephritis score
D.
Anti-nucleosome ab (μg/mL)
E.
Anti-Sm ab (μg/mL)
F.
Anti-RNA ab (μg/mL)
p=0.065
Cybb y/+ donor: 0% 80% 100%
Cybb y/- donor: 100% 20% 0%

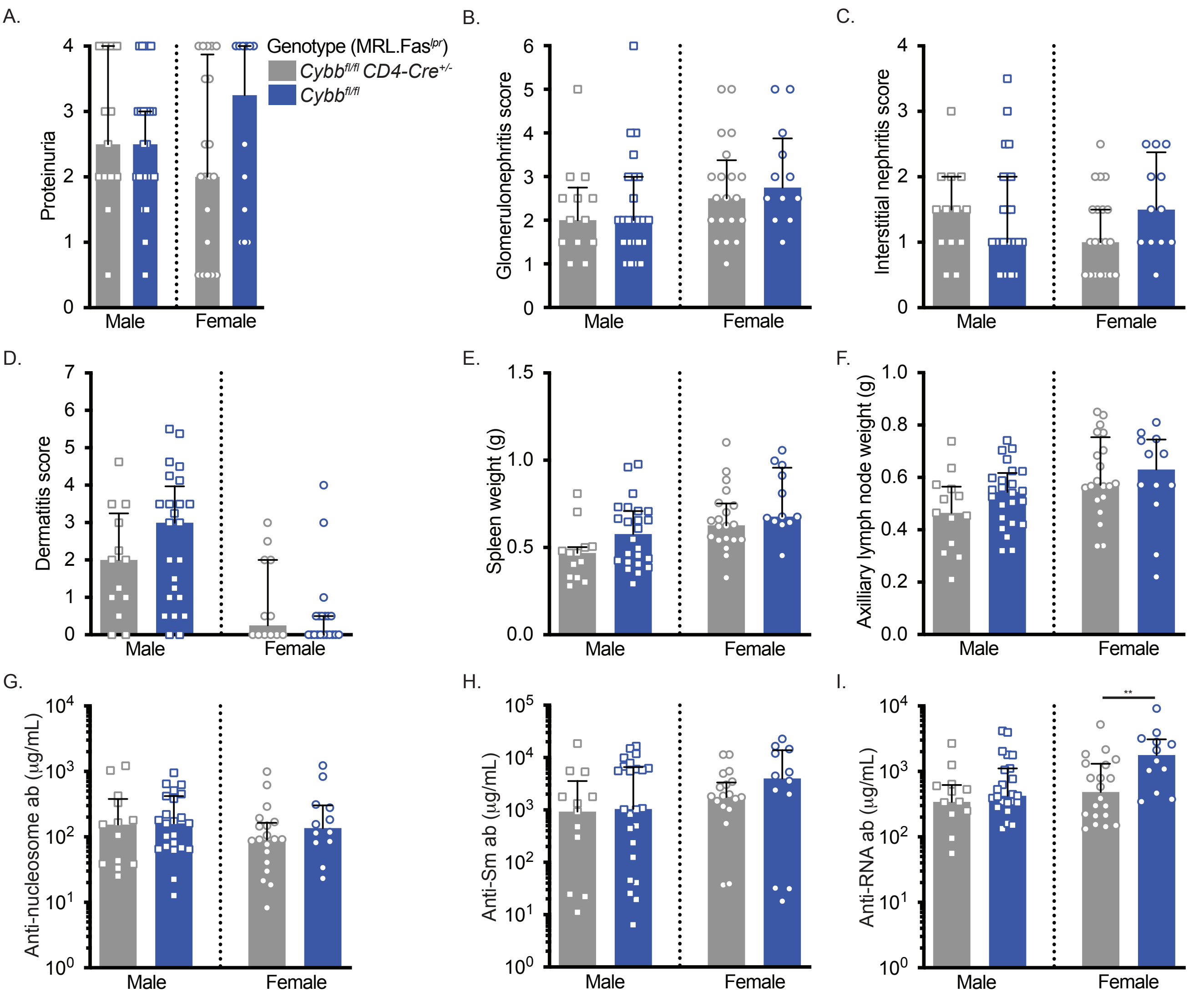

A.
Proteinuria
Genotype (MRL.Fas^lpr)
Cybb^fl/fl CD4-Cre^+/-
Cybb^fl/fl
Male
Female
B.
Glomerulonephritis score
Male
Female
C.
Interstitial nephritis score
Male
Female
D.
Dermatitis score
Male
Female
E.
Spleen weight (g)
Male
Female
F.
Axilliary lymph node weight (g)
Male
Female
G.
Anti-nucleosome ab (μg/mL)
Male
Female
H.
Anti-Sm ab (μg/mL)
Male
Female
I.
Anti-RNA ab (μg/mL)
**
Male
Female
